# Change in quality of malnutrition surveys between 1986 and 2015

**DOI:** 10.1186/s12982-018-0075-9

**Published:** 2018-05-28

**Authors:** Emmanuel Grellety, Michael H. Golden

**Affiliations:** 1 Research Centre Health Policy and Systems - International Health, School of Public Health, Université Libre de Bruxelles, Brussels, Belgium; 2 Department of Medicine and Therapeutics, University of Aberdeen, Aberdeen, Scotland, UK

**Keywords:** Survey, Anthropometry, Nutrition, Child, Weight-for-height, Weight-for-age, Height-for-age, Mid-upper arm circumference, MUAC, Data quality, Standard deviation, Kurtosis

## Abstract

**Background:**

Representative surveys collecting weight, height and MUAC are used to estimate the prevalence of acute malnutrition. The results are then used to assess the scale of malnutrition in a population and type of nutritional intervention required. There have been changes in methodology over recent decades; the objective of this study was to determine if these have resulted in higher quality surveys.

**Methods:**

In order to examine the change in reliability of such surveys we have analysed the statistical distributions of the derived anthropometric parameters from 1843 surveys conducted by 19 agencies between 1986 and 2015.

**Results:**

With the introduction of standardised guidelines and software by 2003 and their more general application from 2007 the mean standard deviation, kurtosis and skewness of the parameters used to assess nutritional status have each moved to now approximate the distribution of the WHO standards when the exclusion of outliers from analysis is based upon SMART flagging procedure. Where WHO flags, that only exclude data incompatible with life, are used the quality of anthropometric surveys has improved and the results now approach those seen with SMART flags and the WHO standards distribution. Agencies vary in their uptake and adherence to standard guidelines. Those agencies that fully implement the guidelines achieve the most consistently reliable results.

**Conclusions:**

Standard methods should be universally used to produce reliable data and tests of data quality and SMART type flagging procedures should be applied and reported to ensure that the data are credible and therefore inform appropriate intervention. Use of SMART guidelines has coincided with reliable anthropometric data since 2007.

**Electronic supplementary material:**

The online version of this article (10.1186/s12982-018-0075-9) contains supplementary material, which is available to authorized users.

## Background

Over the past 30 years, it has become increasingly common to use a single round of anthropometric measurements from a representative sample of children, aged 6–59 months, to assess a population’s nutritional state, particularly in areas thought to be under nutritional stress. These cross-sectional, population-based surveys are used to estimate the prevalence of acute malnutrition, poor growth attainment, and mortality rates as fundamental health indicators. They can then be interpreted with contextual information to plan and implement appropriate interventions. The surveys estimate the scale and type of nutritional intervention required so that personnel with relevant skills, logistics and funds can be requested and mobilised. Where there are many malnourished children programs are implemented to identify and treat the affected children; the planning is mainly based upon data collected by Governments and non-government organisations (NGO). At about 5 yearly intervals either UNICEF’s multiple indicator cluster surveys (MICS) or USA’s Demographic and Health Surveys (DHS) collect information on a large range of health, socio-economic, demographic and environmental variables as well as anthropometric status to give a general overview of a country’s health status [1, 2]. Providing accurate estimates of the prevalence of stunting, wasting and underweight of children is also important for monitoring individual, country and global progress toward the goals of eradicating hunger, reducing health inequalities and assessing the ensuing progress of short- and long-term nutrition and health interventions. Malnutrition prevalence and mortality rate are the primary statistics; when high, the contextual data can be used to interpret the potential causes and indicate which strategic interventions are most appropriate to add to the primary aim of identifying and treating the malnourished.

Thus, in humanitarian emergencies, timely and accurate data are essential to guide decision making by public health care professionals. The survey’s results together with an estimate of the population size show the magnitude and urgency of an affected population’s immediate needs and give a baseline to initiate monitoring the evolution of the emergency and evaluation of the intervention with follow-up surveys. The data also enable governments and United Nations (UN) agencies to properly coordinate the overall response and allows donors to allocate funds appropriately and effectively.

These activities all depend critically upon the accuracy and credibility of the survey data.

There have been guidelines for assessing nutritional status of individuals since the generation of reference values from a healthy population; these have been successively refined from the Baldwin-Wood [3], Harvard [4], NCHS [5], CDC_2000_ [6] and more recently to the WHO_2006_ references [7]. However, the first guideline to propose estimating the prevalence of acute malnutrition of populations from cross-sectional surveys specifically using weight-for-height (WHZ) in Z-scores (standard deviations of a reference population), instead of percent-of-median, was published in 1983 by the World Health Organisation (WHO) [8] to guide the Word Food Program on criteria for admission to supplementary feeding programs. Following further recommendations from WHO in 1989 [9], Epicentre published guidelines for conducting small-scale population based surveys in 1992 [10, 11]. At this same time, survey sampling schemes for use in complex humanitarian emergencies were explored [12–14]. These initiatives were incorporated in an international guideline [15, 16] and used to develop the first software (Epinut) [17] dedicated to analysis of small-scale cross-sectional nutritional surveys. These initiatives were followed in 1995 by WHO’s seminal publication on anthropometry which incorporated and expanded upon all of the earlier works [18].

The application of these survey methods and the accuracy of the data produced by various humanitarian agencies have been criticised [19–24]. Spiegel et al. [19] found gross deficiencies on the basis of failure to use population proportional to size sampling, small sample size, inadequate number of clusters, insufficient number of children per cluster, and non-use of a weight-for-height index. Prudhon and Spiegel [20] analysed reports only and found them to be inadequate. They were sufficiently inadequate for Spiegel to call for surveys to be conducted by professionals only [24], largely because they found that 65% of surveys prior to 2004 were of insufficient quality to be relied upon [20].

In response to this situation the Standardized Monitoring and Assessment of Relief and Transitions (SMART) initiative aimed to simplify and standardize all aspects of conducting a survey, including planning, training, sampling, data collection, analysis, data quality estimation and reporting [25]. Since the introduction of SMART and Emergency Nutrition Assessment (ENA) software [26] in late 2002 many agencies started to adopt these standard methods and most non-governmental organisations (NGOs) and many governments had adopted SMART by 2007. In particular they have used the automatic analysis of the data and the inbuilt facility to statistically check the data quality (“plausibility check”) to provide feedback to the supervisors concerning the performance of the enumerators and an estimate of the credibility of the data for presentation to those relying upon survey results [27].

Several steps are involved in assessing the quality of anthropometric data such as estimating bias in sampling procedures, age and anthropometric errors, and how missing and improbable values are handled. The shape of the distribution (skewness and kurtosis) and the observed standard deviation (SD) of the Z-scores are important statistics that indicate the quality of the data. With accurate age estimates and anthropometric measurements, the SD of the observed distributions should approximate to symmetry when the population is undernourished and have an SD close to the expected value of 1.0 with respect to the reference distribution.

Based on the WHO Technical Report [18], the SD for Weight-for-Height (WFH) should be between 0.8 and 1.2 Z-score units in all well-conducted surveys [18, 28, 29]. This has been confirmed empirically with well conducted surveys in both the developed world where large national surveys of heterogeneous populations have been conducted, for example the National Health and Nutrition Examination Survey (NHANES) from USA’s National Centre for Health Statistics (NCHS) [28] and the developing world [30]. The SD increases substantially as the proportion of random measurement errors in the dataset increases [31]; this has a greater effect upon the prevalence of wasting (WHZ and/or MUAC), underweight (WAZ) and stunting (HAZ) than is usually appreciated. The size of the SD and the number of missing, implausible or flagged subjects give an overall measure of the care with which the enumerators collected and recorded their data, and hence a survey’s credibility. With a single enumeration team a systematic error does not have an effect upon the shape or SD of the distribution; however, with more than one enumeration team, the SD also increases if the various teams have different systematic measurement biases (unpublished: present authors).

The objective of this paper was to examine the change of survey quality over time using the change in the distribution of the anthropometric variables as a criterion of survey reliability, and in particular to determine the quality of surveys since Prudhon’s analysis in April 2004 [20].

## Methods

We performed a secondary analysis of 1843 surveys. The surveys had been conducted in 55 different countries in West Africa (315), Middle Africa (312), East Africa (337), Sahel (657), Northern Asia (60), South Asia (106), Central America (7), Europe (3), Middle East (1) and the Caribbean (45) between 1986 and 2015 (Table 1). Detailed descriptions of the study populations and methods have been published previously [32]. In brief, un-cleaned raw datasets of anthropometric surveys were obtained from 19 agencies working in the field of international nutrition (NGOs, United Nations Agencies and Governments). Data from 11 further agencies that contributed fewer than 5 surveys were also obtained (designated as “other”). The individual survey datasets were initially cleaned by deleting the records of individual children with any of the following criteria: 1) Age < 6 months (n = 26,951), 2) Age > 59 months (n = 11) and 3) Age, sex, weight, or height not recorded (10,610, = 0.74% of data). Children with oedema were also excluded as their anthropometry is affected by the oedema fluid (6748 = 0.47% of data).

**Table 1:** The numbers of surveys, children, oedematous children and percent of subjects flagged using either WHO or SMART flags (excluding oedema), by country

Region	Country	Surveys #	Subjects #	Oedema %	Weight-for-age		Height-for-age		Weight-for-height		MUAC-for-age		MUAC-for-Height	
					WHO	SMART	WHO	SMART	WHO	SMART	WHO	SMART	WHO	SMART
Northern Asia	Afghanistan	55	47,079	0.17	0.10	1.22	0.84	4.15	0.09	1.56	0.03	0.63	0.01	0.41
Europe	Albania	1	906	0.00	0.00	1.32	0.33	5.96	0.00	1.43	0.00	0.22	0.00	0.11
Middle Africa	Angola	45	38,377	0.77	0.03	1.30	0.86	4.95	0.06	1.39	0.03	0.98	0.01	0.89
South Asia	Bangladesh	30	18,407	0.10	0.02	0.39	0.33	1.79	0.01	0.48	0.01	0.11	0.01	0.10
West Africa	Benin	7	7897	0.06	0.00	0.72	0.18	2.15	0.00	0.76	0.00	0.56	0.00	0.29
West Africa	Burkina Faso	67	41,544	0.11	0.02	0.57	0.11	1.44	0.02	0.60	0.01	0.36	0.01	0.24
East Africa	Burundi	32	21,095	1.37	0.09	1.27	1.12	4.04	0.08	1.20	0.02	0.94	0.00	0.68
Middle Africa	Cameroon	9	6034	0.17	0.03	1.18	0.46	3.88	0.08	1.36	0.00	0.60	0.00	0.38
Middle Africa	CAR	49	37,031	0.59	0.01	1.06	0.32	3.52	0.03	0.97	0.01	0.74	0.01	0.57
Middle Africa	Congo-B	1	878	0.46	0.00	0.80	0.00	2.96	0.00	0.91	0.00	0.23	0.00	0.11
Middle Africa	DRC	208	183,478	1.11	0.05	0.79	0.59	2.40	0.04	0.87	0.01	0.79	0.01	0.70
East Africa	Eritrea	2	1628	0.31	0.00	1.29	0.61	3.93	0.25	1.84	0.00	0.12	0.00	0.06
East Africa	Ethiopia	73	64,459	0.23	0.02	0.61	0.23	2.74	0.02	0.67	0.01	0.42	0.00	0.36
West Africa	Gambia	8	6792	0.04	0.01	0.66	0.13	1.62	0.06	0.81	0.00	0.28	0.01	0.16
Latin America	Guatemala	2	1430	0.14	0.21	2.03	1.33	3.99	0.42	1.96	0.00	1.47	0.00	0.98
West Africa	Guinea	17	12,922	0.20	0.02	1.19	0.46	3.10	0.06	1.52	0.02	0.75	0.02	0.58
West Africa	Guinea Bissau	4	2440	0.08	0.04	1.39	0.61	3.16	0.08	0.78	0.00	0.49	0.00	0.33
Caribbean	Haiti	45	36,100	0.41	0.02	1.30	0.39	3.63	0.04	0.89	0.02	0.76	0.01	0.51
South Asia	India	9	5712	0.16	0.23	1.09	1.17	4.11	0.02	0.88	0.00	0.47	0.00	0.26
South Asia	Indonesia	1	394	0.00	0.00	1.02	0.51	5.33	0.00	4.57	0.00	1.52	0.25	1.52
West Africa	Ivory Coast	6	7711	0.74	0.06	1.19	0.43	3.64	0.08	1.23	0.00	0.93	0.00	0.74
East Africa	Kenya	49	33,690	0.30	0.00	0.35	0.19	2.27	0.02	0.51	0.01	0.29	0.00	0.25
Europe	Kosovar	1	921	0.00	0.00	1.41	0.22	2.61	0.11	0.76	0.00	0.33	0.00	0.22
West Africa	Liberia	76	51,361	0.58	0.06	1.51	0.56	4.71	0.08	1.44	0.02	0.83	0.01	0.68
Europe	Macedonia	1	863	0.00	0.00	1.51	0.70	5.91	0.23	1.85	0.12	0.70	0.12	0.60
East Africa	Madagascar	6	4145	0.43	0.02	1.18	0.63	3.93	0.10	0.92	0.02	0.84	0.00	0.51
East Africa	Malawi	34	20,551	1.12	0.07	1.92	1.22	4.82	0.08	1.95	0.02	1.11	0.01	1.00
Sahel	Mali	15	20,060	0.26	0.02	0.86	0.52	4.52	0.16	1.50	0.02	0.47	0.02	0.38
Sahel	Mauritania	51	40,772	0.09	0.03	0.47	0.21	1.85	0.05	0.58	0.00	0.36	0.00	0.29
East Africa	Mozambique	14	3922	2.32	0.05	2.27	0.82	8.36	0.26	1.56	0.05	1.27	0.00	0.98
South Asia	Myanmar	15	11,233	0.17	0.06	0.76	0.66	2.73	0.05	0.56	0.04	0.48	0.00	0.38
South Asia	Nepal	7	4351	0.44	0.09	0.41	0.97	2.39	0.05	0.69	0.02	0.23	0.00	0.21
Latin America	Nicaragua	2	969	0.83	0.00	1.55	0.41	2.27	0.00	0.62	0.00	0.83	0.00	0.42
Sahel	Niger	38	47,284	0.09	0.06	0.82	0.51	3.37	0.06	0.87	0.01	0.45	0.01	0.34
West Africa	Nigeria	45	30,325	0.11	0.04	1.13	0.41	3.43	0.07	1.54	0.02	0.71	0.00	0.57
South Asia	Pakistan	27	24,807	0.13	0.07	0.94	0.50	2.73	0.06	0.97	0.01	0.64	0.00	0.50
South Asia	Philippians	7	3937	0.00	0.03	0.86	0.25	2.90	0.08	0.71	0.00	0.30	0.00	0.20
East Africa	Rwanda	22	12,204	1.26	0.02	1.23	0.57	4.13	0.09	1.52	0.06	0.79	0.04	0.65
Sahel	Senegal	39	30,531	0.09	0.01	0.60	0.18	2.60	0.02	0.51	0.01	0.32	0.01	0.22
West Africa	Sierra Leone	71	57,717	0.41	0.04	1.49	0.45	4.32	0.08	1.52	0.07	1.20	0.02	0.99
Sahel	Somalia	102	78,271	0.78	0.03	1.14	0.46	4.63	0.07	2.56	0.03	0.99	0.02	0.87
Sahel	South Sudan	198	142,796	0.39	0.03	1.23	0.44	5.04	0.14	1.54	0.03	0.70	0.02	0.56
South Asia	Sri Lanka	5	4599	0.02	0.00	0.33	0.07	1.28	0.00	0.37	0.02	0.28	0.00	0.20
Sahel	Sudan	86	72,672	0.15	0.04	0.84	0.29	3.67	0.08	1.01	0.00	0.39	0.00	0.32
Middle East	Syria	1	534	0.19	0.00	0.94	0.75	4.32	0.38	1.69	0.00	0.75	0.00	0.38
Northern Asia	Tajikistan	5	4643	2.30	0.11	2.39	0.80	6.14	0.30	3.79	0.04	1.31	0.00	1.08
East Africa	Tanzania	13	6890	1.38	0.06	1.12	0.70	4.12	0.17	1.65	0.06	1.00	0.06	0.96
Sahel	Chad	128	91,539	0.29	0.03	0.71	0.18	2.87	0.03	0.85	0.02	0.39	0.00	0.30
South Asia	Thailand	2	1813	0.00	0.06	0.77	0.66	2.70	0.06	0.77	0.00	0.11	0.00	0.06
South Asia	Timor	3	1690	0.00	0.06	0.59	0.65	3.96	0.18	0.83	0.00	0.53	0.00	0.30
West Africa	Togo	14	6550	0.56	0.02	0.61	0.15	2.15	0.05	0.52	0.00	0.43	0.00	0.23
East Africa	Uganda	83	52,255	0.33	0.03	1.10	0.35	3.41	0.05	1.11	0.01	0.64	0.00	0.52
Latin America	Venezuela	3	1776	0.17	0.06	0.56	0.06	1.24	0.06	0.23	0.00	0.11	0.00	0.00
East Africa	Zambia	6	3496	0.00	0.00	1.20	0.51	4.66	0.00	1.23	0.00	0.74	0.00	0.49
East Africa	Zimbabwe	3	1361	0.00	0.00	1.18	0.51	5.00	0.00	1.91	0.00	0.73	0.00	0.74
Total	Total	1843	1412,842	0.48	0.04	0.98	0.45	3.49	0.07	1.16	0.02	0.66	0.01	0.53

The number of subjects column is the number of subjects remaining after exclusion of oedema and missing data. WHO flags = all biologically possible data. SMART flags = data that is more than 3.100 SD from the mean of the survey. DRC = Democratic Republic of Congo; CAR = Central African Republic; Congo-B = Congo Brazzaville

Weight-for-height/length (WHZ), height-for-age (HAZ), weight-for-age (WAZ), mid-upper arm circumference (MUAC) for age (MUAC-AgeZ) and height (MUAC-HtZ) indices were calculated in Z-scores using ENA software for SMART [26]. The WHO_2006_ growth standards were used for all calculations. The MUAC-for-age standards and the height-for-age standards were used to derive the MUAC-for-height values. First the “height-age” of each child was calculated (that is the age the child would be if the child was 0.00 Z-score height-for-age); second the MUAC-for-height was calculated using the MUAC-for-age procedure, except that the height-age was substituted for the chronological age. Such a procedure effectively compares the MUAC of the child to that of a child of the same height as the child. The algorithms are incorporated in the ENA software.

In each survey, outliers were excluded using either WHO or SMART flags (Table 1). For SMART flags, children with a HAZ, WAZ, WHZ and MUAC-AgeZ which were more than 3.100 Z-scores above or below the *survey’s mean* were excluded from the analysis of that particular parameter on the basis that a measurement was most likely to be incorrectly measured or recorded, or that they did not properly represent the population being surveyed. Similarly, for absolute MUAC (MUAC-abs), where the MUAC-for-age Z-scores (corrected for any height deficit) were more than 3.100 Z-scores above or below the survey’s mean the absolute MUAC values were flagged and excluded for the analyses using SMART flagging. As WHO recommends retaining all values that are not biologically implausible, the analysis was repeated excluding only those children whose HAZ, WAZ, WHZ or MUAC lay outside the limits considered to be compatible with life specified by WHO [33].

For each survey the mean, standard deviation and moments of kurtosis and skewness were calculated with their 95% CIs [34]. We compared the mean of the SDs of WHZ obtained from each of the 19 different organisations and the combined “other” organisations.

Agency “s” contains the grouped surveys from the 11 “other” organisations. The SD of organisation “t” differs significantly from the others (Student’s *t* test < 0.0001), with 69% (53/77) of their surveys for WHZ having an SD of more than 1.2 Z. This organisation was impaired by gross insecurity; consequently all aspects of their surveys were managed remotely from a separate country. Relatively inexperienced persons were recruited locally, there was no performance testing of enumerators and there was inadequate supervision. It became clear that some of data from some of the surveys had been fabricated as blocks of data were replicated from one survey to another. We therefore omitted this organisation’s data from the main analyses. It became clear from inspection of the individual files that agency “u” had censored their data before we obtained the files. This was shown by quite severe truncation of the tails of the distributions so that each of this agencies’ surveys were unlike any of the other surveys in our database. However, we have included these 8 surveys for completeness (0.5% of surveys).

We then compared the mean SDs and moments of excess kurtosis (kurtosis—3) and skewness of all indices over time using both SMART and WHO flagging procedures. The time periods were chosen to correspond to major advances in survey methodology.

All analyses were performed in R software version 2.9.2 [35].

### Ethics statement

This is a secondary analysis of anonymous data where no individual, cluster or village location could be identified so that formal ethical clearance was not required. Permission to use and analyse the datasets was obtained from the organisations providing the raw datasets.

## Results

**Fig. 1:**
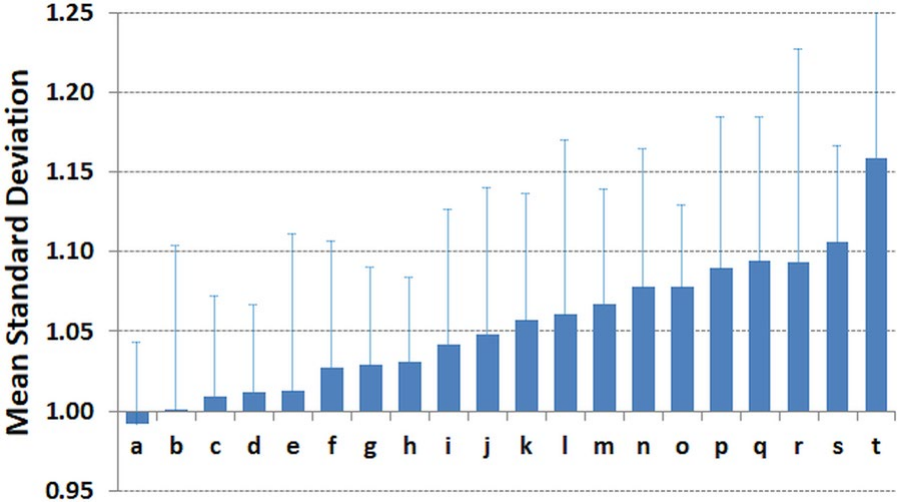
The mean standard deviation in Z-score units of nutritional surveys contributed by different agencies. Agency “a” to “r” and “t” are from single agencies and “agency s” in from 11 different agencies combined that contributed fewer than 5 surveys. The error bars are SDs. The agencies are mainly emergency international non-governmental organisations with 2 governmental and 2 UN agencies

The mean of WHZ SDs of the surveys from each of the agencies is shown in Fig. 1. The numerical data, by agency, are given in Additional file S1. The agencies’ means varied from about 1.00 Z to over 1.15 Z. The two agencies with mean values of over 1.10 were group “s”, which is composed of the 11 agencies contributing fewer than 5 surveys that did not apply or use the SMART guidelines, and agency “t” which had inadequate supervision and has been excluded from the main analysis.

**Fig. 2:**
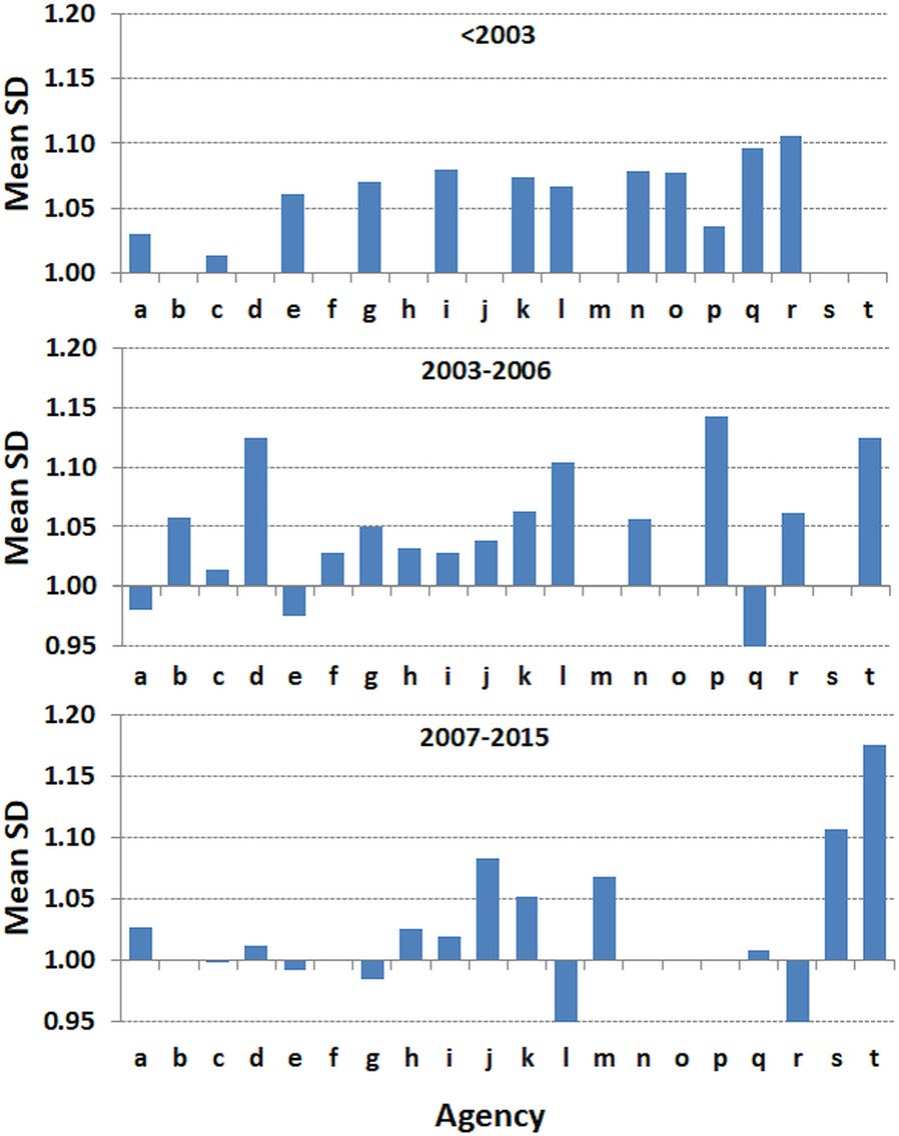
The mean standard deviation in Z-score units of nutritional surveys by time. The surveys that were contributed by the agencies shown in Fig. 1, divided by the date of the surveys: from 1986 to 2002, 2003 to 2006 and 2007 to 2015. The dates mark changes in survey methodology guidance

Although each agency had a different mean SD for WHZ, some contributed surveys before standardisation was introduced and others started to use SMART methods at different intervals (several years) after the guidance was first introduced. The agency data are thus divided by range of years in Fig. 2. Prior to 2003 only 3 of the 12 (25%) contributing agencies had a mean SD of less than 1.05Z and none were at or below 1.00Z. Between 2003 and 2006 when agencies were introducing the standard guidelines 8 of the 16 (50% excluding groups “s” and “t”) agencies had mean values of less than 1.05Z. From 2007, 10 of the 13 (77%) agencies had values less than 1.05 Z of which most were close to 1.00Z. Other agencies that are relatively new to conducting surveys (“j”, “k”, “m”) had slightly higher mean SDs. Two of the agencies had mean values below 0.95 Z. Each of the agencies contributing surveys undertaken in more than one period had a reduction in mean SD except agency “j” and “k”. As the different agencies trained, implemented and became familiar with SMART there was a reduction in the mean SD of their surveys to be close to the expected SD of 1.0Z of the WHO standards.

**Fig. 3:**
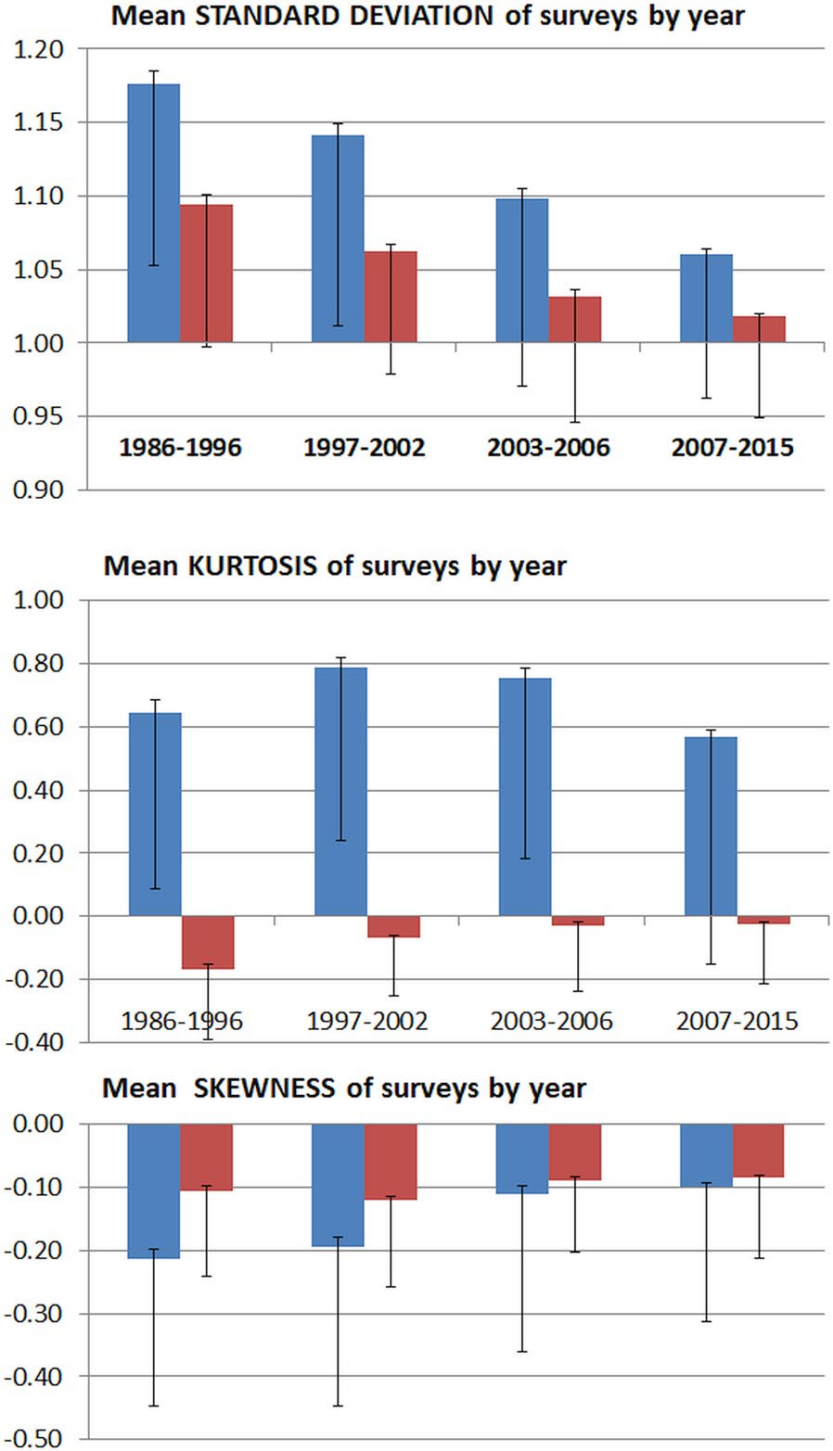
The mean standard deviations, moments of kurtosis and of skewness by date of survey for weight-for-height/length (WHZ). All survey data except for that contributed by agency “t”. The blue columns are for the surveys when applying World Health Organisation criteria for excluding presumably erroneous data points and the red columns using SMART criteria for data exclusion. The error bars above the columns are the standard errors of the mean and below the columns the standard deviations

Figure 3 shows the WHZ distribution from 1766 surveys (excluding agency “t”) conducted between 1986 and 2015 by the 18 single organisations and the 11 other agencies. The data are shown using WHO and SMART flags separately. The SDs have steadily decreased with both flags from around 1.1Z to 1.0Z using SMART flags, as the quality of surveys has improved and the plausibility tests have become more widely used. The mean kurtosis of the distributions are very small and negative using SMART flags; in contrast they are larger and positive when the WHO flags are used to include all biologically plausible data. The mean kurtosis of the data cleaned with SMART flags has been almost zero since 2007. The data, on average, have a slight negative skew, which was initially significantly less with the SMART than with the WHO flags. With time, the skewness has reduced.

With time the discrepancy between the SD, the skewness and, since 1997, kurtosis using the WHO flags and the SMART flags has become steadily smaller. Inclusion of all biologically possible data (WHO procedure), that would be excluded using SMART flags, results in a higher SD, a greater moment of skewness and much larger kurtosis than with the SMART procedure. With a well conducted survey there should be few, if any, data points flagged for removal from a survey. If no data are flagged then the two analyses’ flagging procedures would give identical results. The data shown in Table 2 demonstrates that between 1986 and 2015 the discrepancy between the results obtained with the two flagging procedures has diminished, indicating that with time there are far fewer erroneous data points being recorded in a survey’s dataset that require either form of flagging. Thus, the reduction in the discrepancy is an indication of the improvement of data quality with time. Although there are differences in the mean moments of kurtosis and skewness of the surveys, it is important to consider that with both flagging procedures they are relatively small. Deviations of these moments within plus or minus one unit are generally considered to indicate that the data come from a normally distributed population; this is [36–38] the case with the surveys using SMART flagging, but not for kurtosis using WHO flags as shown by the magnitude of the SD-error bars. For each time period the distribution of the SDs, moments of kurtosis and moments of skewness are much greater when WHO flagging procedures are used than when SMART flags are applied. And with improvements in data quality the results obtained with the two flagging procedures approach one another.

**Table 2:** Change in number of surveys with WHZ SD above 1.2Z over 30 years

Date	Survey	SD > 1.2 WHO	SD > 1.2 SMART	SD > 1.2 WHO	SD > 1.2 SMART	Difference
	#	#	#	%	%	WHO-SMART
1986–1996	212	90	39	42.5	18.4	24.1
1997–2003	325	80	17	24.6	5.2	19.4
2003–2007	364	57	17	15.7	4.7	11.0
2007–2015	865	55	16	6.4	1.8	4.5

All survey data except for that contributed by agency “t”. The dates are selected to mark changes in survey methodology. The difference between WHO and SMART flags give the difference in the WHZ SD when all biologically possible values (WHO) and only values statistically likely to be true values (SMART) are included in the dataset. The differences in the data represent the care with which the enumerators measured height and weight and with an error-free survey should tend to zero

**Fig. 4:**
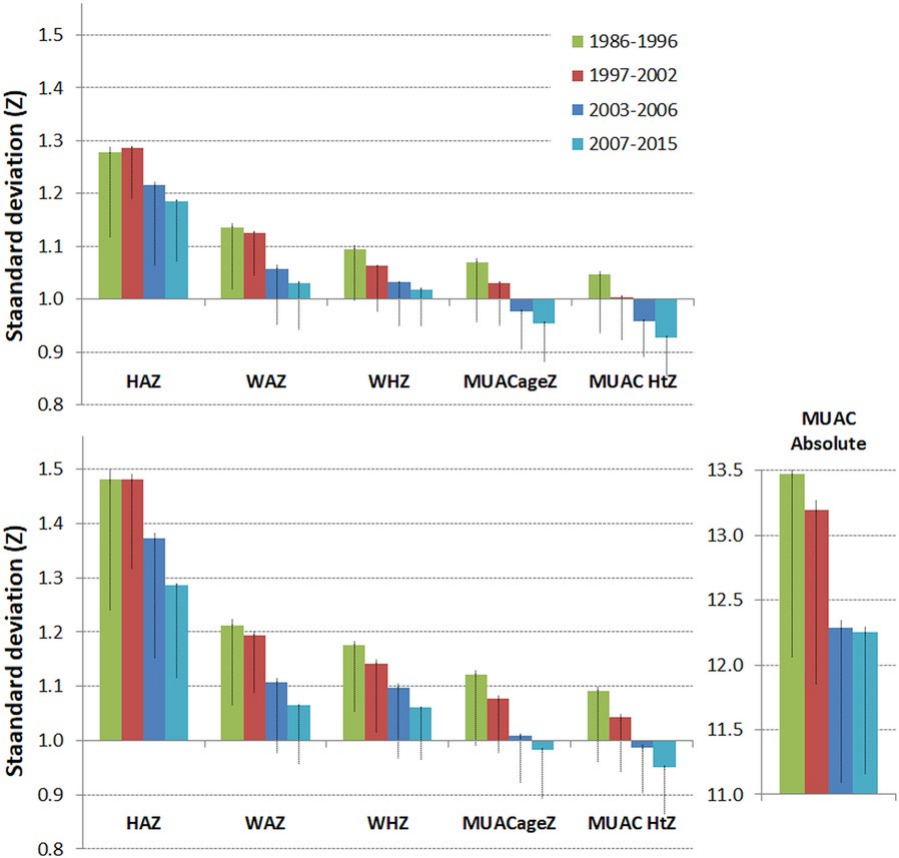
The standard deviations for anthropometric variables by time. All survey data except for that contributed by agency “t”. The standard deviations of height-for-age (HAZ), weight-for-age (WAZ), weight-for-height/length (WHZ), MUAC-for-age (MUACageZ), MUAC-for-height (MUAC HtZ) each in Z-score units and absolute MUAC in mm, by date of conducting the survey. The upper panel shows the data applying SMART flags and the lower panel with WHO flags. The error bars above the columns are the standard errors of the mean and below the columns the standard deviations

Figure 4 shows the corresponding mean SDs for all the anthropometric variables using SMART flags in the upper panel and WHO flags in the lower panel. The absolute MUAC is shown in the lower panel (units are mm). These data confirm the observations reported for WHZ with each of the other variables. The SDs of each variable have reduced over the time-span that these data have been collected. With each of the variables the results of using the SMART flagging procedure results in a lower SD than with WHO flag excluded data. The highest SDs are for height-for-age, and the reduction in their SDs with time is most marked when WHO flags are applied (from 1.5 Z to 1.3 Z). The mean MUAC-ageZ and MUAC-htZ SDs are now less than the WHO standards. There has also been a marked reduction in the absolute MUAC’s SDs from 13.5 to 12.2 mm (11%).

**Fig. 5:**
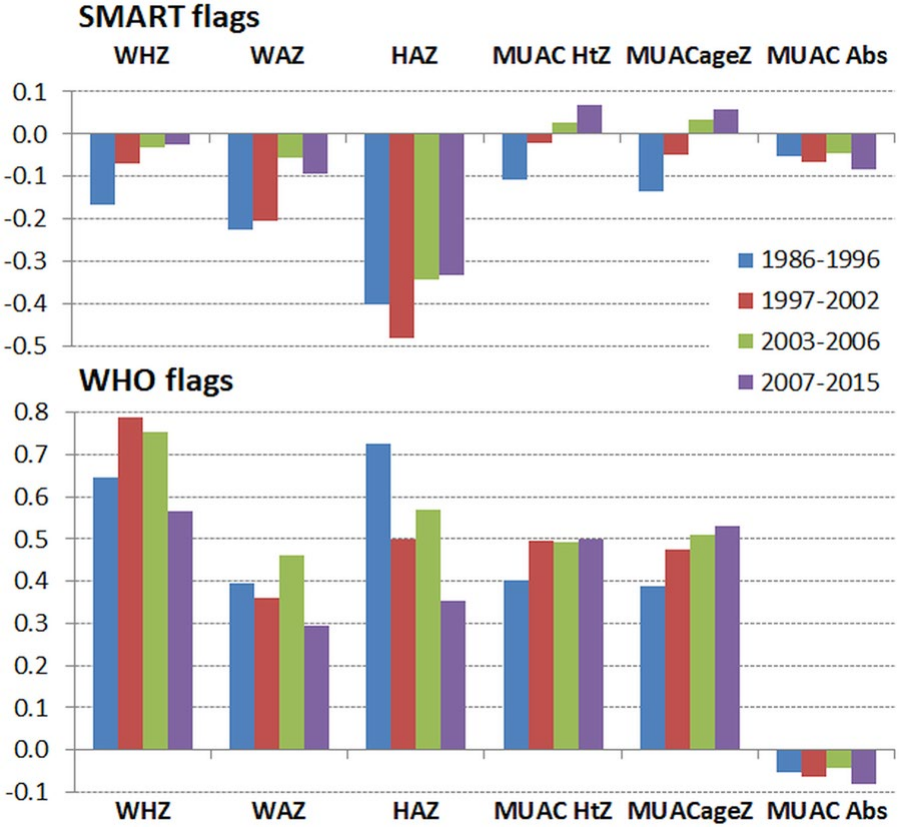
The moments of kurtosis for anthropometric variables by time. All survey data except for that contributed by agency “t”. The kurtosis of height-for-age (HAZ), weight-for-age (WAZ), weight-for-height/length (WHZ), MUAC-for-age (MUACageZ), MUAC-for-height (MUAC HtZ) and absolute MUAC (MUAC Abs), by date of conducting the survey. The upper panel shows the data applying SMART flags and the lower panel with WHO flags

The corresponding change in kurtosis is shown in Fig. 5. In terms of data quality the moment of kurtosis is a measure of the amount of data that is in the tails of the distribution relative to a Gaussian distribution; the shape of the central portion of the distribution has a negligible effect upon the moment of kurtosis [39]. A positive kurtosis indicates that the tails are long and contain outliers, a negative kurtosis indicates that the tails are shorter than expected compared to a Gaussian distribution. Using WHO flags there is a much larger and positive kurtosis for all the anthropometric variables (except absolute MUAC) indicating longer tails and excess outliers. There has been a reduction in WHZ, WAZ and HAZ kurtosis between 1997 and post-2007 using both flagging procedures indicating a general increase in the quality of data. Using SMART flags there was initially a small negative kurtosis with a reduction in the kurtosis of WHZ, WAZ to almost zero after 2007; the kurtosis for each of the MUAC variables is close to zero when SMART flagging is applied.

**Fig. 6:**
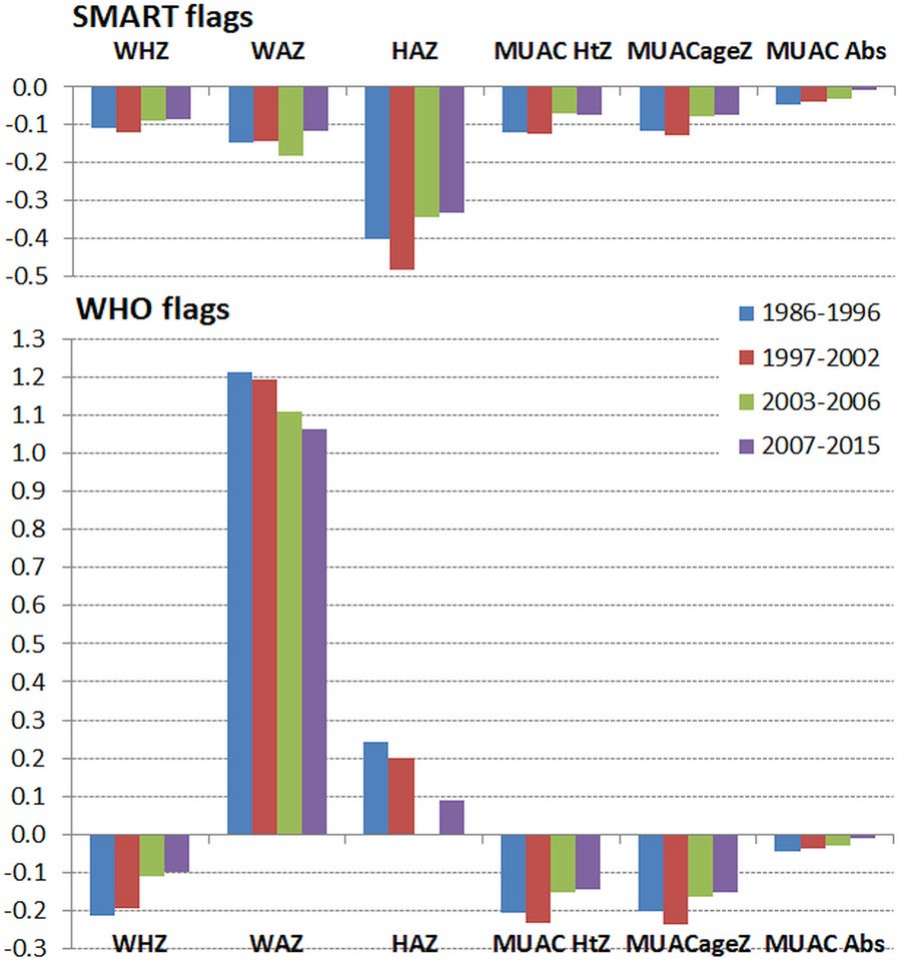
The moments of skewness for anthropometric variables by time. All survey data except for that contributed by agency “t”. The skewness of height-for-age (HAZ), weight-for-age (WAZ), weight-for-height/length (WHZ), MUAC-for-age (MUACageZ), MUAC-for-height (MUAC HtZ) and absolute MUAC (MUAC Abs), by date of conducting the survey. The upper panel shows the data applying SMART flags and the lower panel with WHO flags

The mean moments of skewness are shown in Fig. 6. Positive skewness indicates that the right tail of the distribution is longer or fatter than expected with a Gaussian distribution whereas a negative skewness indicates that the left tail is similarly asymmetric. However moments of skewness can be difficult to interpret because a long tail on one side can be balanced by a fat, but shorter, tail on the other side. Nevertheless, it is an indication of the symmetry of the data. With SMART flags there is a slight negative skewness with a marginal reduction with time for most of the variables; there is a larger negative skew with HAZ. This indicates that using SMART flags there is slightly more of a tail on the malnourished end of the distribution; this indicates that the flagging procedure has not stripped excess negative (i.e. malnourished children) values from the dataset relative to positive values. The skewness using WHO flags has generally lessened with time; however, including all biologically possible data has resulted in a large positive skew (relatively more high weights) in the WAZ data.

The plots of the individual survey’s SD by the actual date the survey was performed for each of the anthropometric variables and flagging procedures are shown in Supplementary File 2. They confirm the summary data presented and show that since 2007 there has not been a further reduction in shape or spread of the distribution the anthropometric variables of interest. The supplementary material also shows that there has been no change in the mean age or height of the subjects included in the surveys over the time-span of the data reported. This indicates that the children, during the time span of the surveys, have not changed and would not account for any change in the distribution of the variables (n.b. a random error of measurement does not alter the mean value of a variable, only its statistical distribution). There has been a slight reduction in the mean absolute weight of the children surveyed.

## Discussion

Our results show that there has been an improvement in the distributions of anthropometric variables used to assess the prevalence of malnutrition over the past 30 years. Since 2007 the surveys have a mean SD for WHZ, WAZ, MUAC-ageZ and MUAC-HtZ variables which are close to the theoretical of 1.00Z with a small standard deviation of about 0.05Z as well as tiny moments of kurtosis and skewness. This is in contrast to the analysis of surveys prior to 2004 which were heavily criticised by Prudhon and Spiegel [20].

HAZ always has a higher SD in such surveys because of the errors inherent in age estimation; even an error of 2 months can increase the SD from 0.0Z to 1.45Z and a random error of 3 months to about 2.0Z [31]. Nearly all surveys show age “heaping” at 6 month intervals.

As survey protocols, training, cluster sampling schemes, cleaning criteria and feedback using the plausibility check, have been progressively taken up by agencies there has been a marked improvement in data quality. In the past survey managers had no method of assessing the data quality until after the survey was completed and analysed, and there was no standard report format with space indicated for essential narrative information to be incorporated. As the software analyses and “writes” the basic report, analytical and clerical errors are reduced and essential information is prompted [26]. Also the automatic data quality procedures, built into the ENA software, has enabled survey managers to check on both the overall quality and the performance of each of the enumeration teams separately as the survey is in progress. They can identify any under-performance, need for re-training, changing or additional supervision to correct defects and errors [27]. Prior to the introduction of SMART in 2002 many surveys had a higher than acceptable SD. As agencies began to adopt and become familiar with these standardised methods to conduct a nutritional survey between 2003 and 2007 there was an improvement in data quality. Since 2007, most agencies that used the guidance provided by SMART produce surveys whose data quality has stabilised with a mean SD of close to 1.00 Z scores. The agencies that have not followed this guidance (e.g. group “s”) or have not been able to be supervised because of major security concerns (e.g. group “t”) have remained with a high SD. This is also clearly shown by comparison of the data obtained by 3 different survey protocols at approximately the same time in countries in West Africa [31]. The reduction in SD over time by the agencies that implemented SMART methods and the failure to reduce the SD when older methods have persisted or supervision has been poor, confirms that the SD is a useful measure of the care with which the teams have collected and recorded the measurements. This is reinforced by the different performance of the various agencies. Despite adequate guidelines, nutrition surveys are still not always done with methodological rigour by some agencies; and “remote” supervision from head-quarters nearly always leads to poor quality data (this was the problem with agency “r”). Our data demonstrate that agencies get different mean SD values from others. Such differences cannot be ascribed to variations in the populations being surveyed but reflect upon the degree of staff training, supervision and application of recommended methods.

The WHO standards are based upon centiles which have been converted into Z-scores; these are different from the use of the term Z-score in the statistical literature because the numerical difference between 0 and − 1Z is not the same as between − 1 and − 2Z etc. In this way the slight positive skewness of most populations has been “normalised” to a Gaussian distribution using the LMS (lambda-mu-sigma) procedure [40] and the data are expected to be normally distributed. The reference population has been selected from 6 populations from different continents; an undoubtedly heterogeneous sample. It is expected that a similarly heterogeneous population in a sample will also approximate to a normal Gaussian distribution. Indeed, large heterogeneous samples taken with stringent control of data quality, such as the NHANES surveys of the USA by the NCHS, approximate to a Gaussian distribution with a standard deviation of 1.0 Z [18, 28]. An ethnically homogeneous population may thus have a lower SD than the WHO standards. Most of the surveys included in the present analysis were from ethnically homogeneous populations and yet their SDs approximated to 1.00Z. It would appear that the homogeneity of the population does not have a marked effect upon the anthropometric parameters considered in assessing malnutrition. This accords with the similarity of the different data-sets that were combined by WHO in establishing their standards.

There are always random measurement errors and this always leads to an increase in the SD of a variable [31]. In other words, unless errors are all trivial, the SD is always greater than the *true SD* of the population. Importantly the effect is not reduced by an increase in sample size, as is sometimes assumed [31]. Where several enumeration teams are used and one or more of the teams has a systematic bias in a measurement, this will also result in an increase in the “spread” of the data and hence an increase in the SD. For most anthropometric measurements the SD from single surveys should lie between 0.8 and 1.2 [18], with about 80% between 0.9 and 1.1Z [30]. For these reasons the SD has been used as a useful measurement of data quality [25, 29, 41]. We have used this parameter to examine whether there has been a change in the quality of surveys with the introduction of standardised methods of conducting surveys. Prior to 1992 there were relatively few anthropometric surveys performed. With the introduction of Epi-info incorporating Epi-nut software, many more small-area surveys were performed to assess individual population’s nutritional status; however, there were few epidemiologists available to be involved, the command-line driven software required specific expertise to use, sampling often used “spin-the-pen” [42] and other biased sampling methods and it was thought that random error would not affect the results provided a sufficient sample size was achieved [31]. In late December 2002 SMART guidelines and software was introduced at an international conference organised by the United States Agency for International Development (USAID) in Washington. SMART specifically addressed the gaps left by previous guidelines and was designed to allow non-epidemiologists to sample, train, acquire, analyse and report high quality nutritional data. Aspects such as the sampling frame, sample size calculation, data quality assessment and reporting were all automated in the software, without the user having to understand the mathematics or use the formulae involved. This was meant to address the severe criticisms of surveys that were being reported and used to guide policy and humanitarian intervention [19–24].

Corsi et al. [41] and Grellety and Golden [31] examined the differences in distribution between MICS, DHS and National Government surveys (using SMART), each of which used different guidelines and methods (all using WHO flags). They report that the SDs of the MICS and DHS surveys were substantially higher than the National Government surveys and frequently outside the acceptable range.

It appears that the precision with which MUAC is taken has also improved. Frison et al. [43] suggest that statistical approaches relying on the normal distribution assumption can be successfully applied to MUAC. However, we have no data to explain why the mean SDs for the MUAC variables are less than one. We speculate that this is due to ethnic differences in fat patterning as usually our surveys individually came from ethnically homogeneous populations whereas the standards were deliberately derived from heterogeneous populations. There are greater ethnic differences on body fat distribution than in other anthropometric measurements [32, 44].

There is controversy over which flags should be used to avoid bias caused by including erroneous data in the analysis. The recommended WHO procedure makes the assumption that all data that is compatible with life and biologically possible has been accurately taken and should be included in the analysis. The proportion of the data excluded because they are not possible measurements is also a good indicator of data quality. It is likely that the higher the proportion of impossible data the higher the proportion of erroneous measurements that are included in the dataset. However, it is an unrealistic assumption that there are no errors of measurement; there are always errors. If these are minimised and trivial it will make little difference to the results. However, as we have shown, the greater the error the wider the SD and the higher the prevalence of abnormality shown by the analysis [31]. Survey SDs are therefore always overestimated to a greater or lesser extent; and that extent determines the level of over-estimation of the prevalence of malnutrition. This is a statistical reality. Indeed, the consistently high SD for HAZ because of errors of age estimation are testimony to the difficulty in obtaining accurate data and the effect of inaccurate data. As the data has improved in quality the results obtained with WHO-flagging and SMART-flagging have converged. This itself is an indication that the results using SMART flagging are more accurate than those which incorporate all biologically possible measurements.

SMART flags use statistical probability to assess whether data points are likely to be erroneous or accurate. The flags are based on the observed population mean as opposed to the mean of the standards. They are set at a fixed number of standard deviations from this mean. Theoretically, if the SD is 1.00Z then one true data point will be outside ± 3.10 Z from the mean out of a sample of 1000 subjects. Such individuals undoubtedly exist and are remarked upon by enumerators, but the false exclusion of 1/1000 of the dataset would have a trivial effect upon a reported prevalence. However, there are normally many more than 1/1000 subjects outside this limit in less than well-done surveys and statistically most of these are errors. The SMART procedure assumes that these values are errors and should be excluded. Inclusion of such erroneous values will generate a positive kurtosis (see Fig. 5) as seen when only WHO flags are used and the outliers are included in the analysis. The fact that with training the mean SDs over the years using the two flagging procedures have converged indicates the improvement of data collection. Furthermore, the post 2007 data for SD, kurtosis and skewness indicate that using a cut-off point of ± 3.100Z is appropriate, rather than using wider “windows-of-inclusion”.

Crowe et al. [45] reported that the effect of survey’ cleaning criteria on the estimation of wasting prevalence with WHO flags criteria is more ‘inclusive’ (and thus tend to give higher prevalence results) whereas SMART flags are more ‘exclusive’ (and thus give lower prevalence results). We agree with Crowe’s analysis; the question to be answered is which gives a more reliable estimate of the true prevalence? Our theoretical analysis [31] and the present data indicate that using SMART flags results in a more reliable estimate and the WHO procedure consistently over-estimates the prevalence unless the survey is of very high quality.

A limitation of this study is that most of the studies come from stressed African populations and children from Latin America, South and South-East Asia are relatively under represented.

Furthermore, the surveys included were not classified by other measures of data quality apart from the shape of the distribution. This was because for many of the studies we did not have access to the full narrative report; it is on the basis of such narrative reports that others have criticised the quality of surveys [19–24]; to our knowledge this is the first report to examine such a large number of surveys using the internal structure of the numerical data to assess the actual quality of the data analysed.

## Conclusion

Weight, height and MUAC based anthropometric indicators are used worldwide to characterize the nutritional status of populations. This study shows that the mean SD of these parameters approximate to the distribution of the WHO standards coincidentally with the introduction and uptake of simplified survey guidelines, automatic data quality checks and software have been introduced and implemented. Those agencies that have not followed the guidelines obtain inflated prevalence figures from anthropometric surveys. The results also show that exclusion of data based upon SMART flag cut-off points, rather than including all data that is compatible with life further improves data quality of anthropometric surveys. Agencies vary in their uptake and adherence to standard guidelines; this is reflected in the mean SD values of the surveys they contributed; those agencies that have fully embraced SMART achieve distributions of survey data similar to the WHO standards. Standardization tests [46] should be performed and reported systematically to confirm the ability of the staff to perform sufficiently precise and accurate measurements. Analysis of the quality of anthropometric data is only a subset of the information that is needed to assess the overall quality of a population-based survey. Well-defined and internationally accepted criteria to assess survey quality should be universally applied and reported if the surveys are to be reliable, credible and form the basis for appropriate intervention and command donor support [20].

## Additional files

**Additional file 1.** Details of the surveys by agency.

**Additional file 2.** Plots of individual survey’s anthropometric variables. All survey data except for that contributed by agency “t”. XY plots of the individual survey SDs against the actual date of the survey for respectively: WHZ, HAZ, MUAC-for-age, WAZ, MUAC-for-height, absolute MUAC (each applying SMART and WHO flags), chronological age, absolute weight and absolute height. The polynomial regression lines are shown in red.

## Abbreviations

DHS: demographic and health surveys
ENA: emergency nutrition assessment
HAZ: height-for-age Z-score
MICS: multiple indicator cluster surveys
MUAC: mid-upper arm circumference
MUAC-AgeZ: MUAC for age z-score
MUAC-HtZ: MUAC for height Z-score
NCHS: National centre for health statistics
NGO: non-government organisations
NHANES: National health and nutrition examination survey
SD: standard deviation
SMART: standardized monitoring and assessment of relief and transitions
UN: United nations
USAID: United states agency for international development
WAZ: weight-for-age Z-score
WHO: World health organization
WFH: weight-for-height
WHZ: weight-for-height Z-score
