# Supplementary material for: Change in quality of malnutrition surveys between 1986 and 2015
Source: Emerg Themes Epidemiol. 2018 May 28;15:8. doi: 10.1186/s12982-018-0075-9 (PMC5972441; doi:10.1186/s12982-018-0075-9)
Supplement: Supplementary file 1 — Additional file 1. Details of the surveys by agency. [file 12982_2018_75_MOESM1_ESM.docx]

**Additional File S1.** The characteristics of the surveys contributed by different agencies

| **agency** | **dates** | **surveys** | **subjects** | **WHZ Standard deviations** | | | | **WHZ Kurtosis** | | | | **WHZ Skewness** | | | |
| --- | --- | --- | --- | --- | --- | --- | --- | --- | --- | --- | --- | --- | --- | --- | --- |
|  |  |  |  | **SMART** | **WHO** | **SMART** | **WHO** | **SMART** | **WHO** | **SMART** | **WHO** | **SMART** | **WHO** | **SMART** | **WHO** |
|  |  | # | # | mean | mean | **±95% CI** | **±95% CI** | mean | mean | **±95% CI** | **±95% CI** | mean | mean | **±95% CI** | **±95% CI** |
| a | 1999-2014 | 45 | 38,476 | 0.992 | 1.032 | 0.977-1.007 | 1.010-1.053 | 0.006 | 0.591 | -0.038, 0.051 | 0.452, 0.730 | -0.058 | -0.035 | -0.093, -0.023 | -0.088, 0.018 |
| b | 2001-2004 | 6 | 4,475 | 1.001 | 1.083 | 0.918-1.083 | 0.953-1.212 | 0.134 | 1.293 | -0.045, 0.314 | 0.732, 1.854 | -0.083 | -0.088 | -0.140, -0.026 | -0.313, 0.137 |
| c | 2000-2011 | 43 | 33,660 | 1.009 | 1.060 | 0.991-1.028 | 1.037-1.082 | -0.009 | 0.753 | -0.057, 0.039 | 0.577, 0.930 | -0.074 | -0.088 | -0.114, -0.034 | -0.154, -0.022 |
| d | 2005-2014 | 378 | 273,255 | 1.012 | 1.060 | 1.007-1.018 | 1.053-1.068 | 0.000 | 0.696 | -0.017, 0.017 | 0.634, 0.758 | -0.078 | -0.110 | -0.089, -0.066 | -0.131, -0.089 |
| e | 2000-2014 | 35 | 29,036 | 1.013 | 1.079 | 0.980-1.045 | 1.032-1.126 | 0.004 | 0.861 | -0.064, 0.073 | 0.633, 1.089 | -0.056 | -0.100 | -0.098, -0.013 | -0.170, -0.031 |
| f | 2005-2006 | 10 | 9,007 | 1.028 | 1.108 | 0.979-1.077 | 1.022-1.194 | -0.031 | 0.805 | -0.115, 0.054 | 0.469, 1.142 | -0.031 | -0.004 | -0.099, 0.038 | -0.179, 0.171 |
| g | 2000-2008 | 15 | 27,940 | 1.034 | 1.105 | 1.001-1.067 | 1.056-1.155 | -0.059 | 0.804 | -0.114, -0.005 | 0.614, 0.995 | -0.123 | -0.151 | -0.162, -0.084 | -0.230, -0.072 |
| h | 2005-2012 | 15 | 14,039 | 1.031 | 1.103 | 1.005-1.058 | 1.056-1.150 | -0.033 | 0.951 | -0.086, 0.020 | 0.706, 1.197 | -0.022 | 0.015 | -0.076, 0.031 | -0.131, 0.161 |
| i | 1992-2014 | 925 | 706,503 | 1.042 | 1.097 | 1.037-1.048 | 1.089-1.104 | -0.065 | 0.579 | -0.078, -0.052 | 0.535, 0.622 | -0.103 | -0.135 | -0.111, -0.094 | -0.150, -0.120 |
| j | 2000-2010 | 13 | 11,099 | 1.053 | 1.169 | 1.003-1.104 | 1.095-1.242 | -0.073 | 1.346 | -0.182, 0.035 | 1.055, 1.636 | -0.095 | -0.083 | -0.153, -0.036 | -0.231, 0.066 |
| k | 2001-2010 | 28 | 26,096 | 1.057 | 1.148 | 1.028-1.086 | 1.105-1.190 | -0.064 | 1.052 | -0.119, -0.010 | 0.832, 1.272 | -0.100 | -0.104 | -0.137, -0.063 | -0.177, -0.031 |
| L | 1992-2013 | 47 | 34,003 | 1.060 | 1.128 | 1.029-1.092 | 1.091-1.165 | -0.130 | 0.706 | -0.193, -0.066 | 0.489, 0.922 | -0.161 | -0.401 | -0.199, -0.123 | -0.468, -0.334 |
| m | 2012-2014 | 7 | 3,973 | 1.068 | 1.088 | 1.015-1.120 | 1.022-1.154 | -0.027 | 0.119 | -0.260, 0.205 | -0.202, 0.439 | -0.046 | -0.081 | -0.171, 0.079 | -0.226, 0.064 |
| n | 1992-2005 | 50 | 36,005 | 1.078 | 1.151 | 1.054-1.102 | 1.120-1.181 | -0.143 | 0.653 | -0.203, -0.082 | 0.514, 0.791 | -0.105 | -0.22 | -0.140, -0.069 | -0.269, -0.172 |
| o | 2000-2000 | 11 | 8,607 | 1.078 | 1.151 | 1.047-1.108 | 1.091-1.211 | -0.076 | 0.731 | -0.170, 0.019 | 0.458, 1.003 | -0.136 | -0.202 | -0.170, -0.102 | -0.313, -0.091 |
| p | 2001-2007 | 6 | 4,730 | 1.089 | 1.239 | 1.013-1.166 | 1.064-1.414 | -0.087 | 0.768 | -0.219, 0.045 | 0.393, 1.143 | -0.116 | -0.220 | -0.179, -0.053 | -0.418, -0.022 |
| q | 1986-2010 | 47 | 21,072 | 1.094 | 1.176 | 1.068-1.120 | 1.146-1.206 | -0.147 | 0.632 | -0.222, -0.072 | 0.487, 0.778 | -0.122 | -0.270 | -0.161, -0.083 | -0.331, -0.209 |
| r | 1998-2005 | 62 | 52,347 | 1.094 | 1.219 | 1.060-1.127 | 1.162-1.276 | -0.072 | 0.888 | -0.136, -0.008 | 0.740, 1.036 | -0.094 | -0.072 | -0.130, -0.059 | -0.161, 0.017 |
| s | 2012-2014 | 15 | 7,638 | 1.107 | 1.209 | 1.075-1.138 | 1.117-1.300 | -0.192 | 0.559 | -0.291, -0.094 | 0.245, 0.873 | -0.056 | -0.024 | -0.125, 0.013 | -0.116, 0.068 |
| t | 2003-2011 | 77 | 61,788 | 1.159 | 1.285 | 1.134-1.183 | 1.248-1.321 | -0.192 | 0.633 | -0.251, -0.133 | 0.505, 0.762 | 0.001 | 0.169 | -0.036, 0.038 | 0.112, 0.226 |
| u | 1993-1999 | 8 | 9,093 | 0.937 | 0.985 | 0.920-0.953 | 0.951-1.018 | 0.145 | 1.258 | 0.017, 0.274 | 0.772, 1.744 | -0.092 | -0.142 | -0.167, -0.016 | -0.309, 0.025 |

WHZ weight-for-height Z-score; SMART, results when SMART flagging procedures are applied; WHO, results when biologically impossible data are excluded.

| **agency** | **dates** | **surveys** | **subjects** | **HAZ Standard deviations** | | | | **HAZ Kurtosis** | | | | **HAZ Skewness** | | | |
| --- | --- | --- | --- | --- | --- | --- | --- | --- | --- | --- | --- | --- | --- | --- | --- |
|  |  |  |  | **SMART** | **WHO** | **SMART** | **WHO** | **SMART** | **WHO** | **SMART** | **WHO** | **SMART** | **WHO** | **SMART** | **WHO** |
|  |  | # | # | mean | mean | **±95% CI** | **±95% CI** | mean | mean | **±95% CI** | **±95% CI** | mean | mean | **±95% CI** | **±95% CI** |
| a | 1999-2014 | 45 | 38,476 | 1.211 | 1.341 | 1.177-1.246 | 1.286-1.397 | -0.429 | 0.228 | -0.497, -0.361 | 0.110, 0.346 | -0.026 | -0.056 | -0.062, 0.010 | -0.114, 0.003 |
| b | 2001-2004 | 6 | 4,475 | 1.352 | 1.547 | 1.270-1.434 | 1.443-1.651 | -0.567 | 0.198 | -0.739, -0.395 | -0.247, 0.642 | 0.119 | 0.202 | 0.064, 0.174 | 0.060, 0.344 |
| c | 2000-2011 | 43 | 33,660 | 1.286 | 1.450 | 1.267-1.305 | 1.422-1.478 | -0.470 | 0.471 | -0.518, -0.422 | 0.309, 0.633 | 0.103 | 0.245 | 0.073, 0.132 | 0.176, 0.313 |
| d | 2005-2014 | 378 | 273,255 | 1.164 | 1.276 | 1.154-1.173 | 1.262-1.291 | -0.266 | 0.591 | -0.286, -0.245 | 0.534, 0.648 | 0.064 | 0.117 | 0.052, 0.076 | 0.095, 0.139 |
| e | 2000-2014 | 35 | 29,036 | 1.198 | 1.341 | 1.158-1.238 | 1.275-1.406 | -0.371 | 0.528 | -0.456, -0.285 | 0.309, 0.747 | -0.005 | 0.052 | -0.043, 0.033 | -0.042, 0.147 |
| f | 2005-2006 | 10 | 9,007 | 1.290 | 1.496 | 1.241-1.339 | 1.414-1.579 | -0.547 | 0.363 | -0.623, -0.471 | 0.079, 0.647 | 0.013 | 0.113 | -0.040, 0.066 | -0.034, 0.260 |
| g | 2000-2008 | 15 | 27,940 | 1.265 | 1.435 | 1.226-1.304 | 1.368-1.502 | -0.437 | 0.684 | -0.531, -0.343 | 0.354, 1.013 | 0.117 | 0.345 | 0.075, 0.158 | 0.196, 0.495 |
| h | 2005-2012 | 15 | 14,039 | 1.180 | 1.317 | 1.109-1.251 | 1.211-1.422 | -0.292 | 0.419 | -0.420, -0.165 | 0.196, 0.642 | -0.052 | -0.113 | -0.102, -0.001 | -0.200, -0.026 |
| i | 1992-2014 | 925 | 706,503 | 1.217 | 1.347 | 1.208-1.227 | 1.333-1.361 | -0.364 | 0.397 | -0.402, -0.326 | 0.329, 0.465 | 0.008 | 0.068 | -0.004, 0.019 | 0.048, 0.089 |
| j | 2000-2010 | 13 | 11,099 | 1.349 | 1.578 | 1.292-1.406 | 1.492-1.663 | -0.602 | 0.269 | -0.710, -0.493 | 0.056, 0.482 | 0.090 | 0.168 | 0.028, 0.151 | 0.062, 0.275 |
| k | 2001-2010 | 28 | 26,096 | 1.312 | 1.515 | 1.272-1.352 | 1.455-1.576 | -0.547 | 0.401 | -0.616, -0.478 | 0.168, 0.633 | 0.073 | 0.170 | 0.046, 0.100 | 0.108, 0.233 |
| L | 1992-2013 | 47 | 34,003 | 1.325 | 1.522 | 1.289-1.361 | 1.465-1.579 | -0.552 | 0.488 | -0.625, -0.478 | 0.218, 0.759 | 0.082 | 0.274 | 0.049, 0.115 | 0.185, 0.363 |
| m | 2012-2014 | 7 | 3,973 | 1.242 | 1.281 | 1.181-1.302 | 1.203-1.359 | -0.467 | -0.276 | -0.581, -0.354 | -0.446, -0.106 | 0.058 | 0.014 | -0.025, 0.141 | -0.085, 0.112 |
| n | 1992-2005 | 50 | 36,005 | 1.305 | 1.521 | 1.273-1.337 | 1.474-1.569 | -0.477 | 0.888 | -0.561, -0.394 | 0.570, 1.206 | 0.103 | 0.384 | 0.072, 0.134 | 0.283, 0.486 |
| o | 2000-2000 | 11 | 8,607 | 1.331 | 1.508 | 1.277-1.385 | 1.442-1.575 | -0.650 | 0.324 | -0.788, -0.513 | 0.038, 0.609 | 0.080 | 0.244 | 0.026, 0.134 | 0.169, 0.319 |
| p | 2001-2007 | 6 | 4,730 | 1.364 | 1.612 | 1.322-1.405 | 1.483-1.741 | -0.684 | 0.169 | -0.787, -0.581 | -0.049, 0.387 | 0.018 | 0.185 | -0.031, 0.068 | 0.082, 0.288 |
| q | 1986-2010 | 47 | 21,072 | 1.280 | 1.482 | 1.249-1.311 | 1.429-1.534 | -0.437 | 0.758 | -0.516, -0.358 | 0.487, 1.030 | 0.096 | 0.342 | 0.066, 0.126 | 0.255, 0.429 |
| r | 1998-2005 | 62 | 52,347 | 1.287 | 1.555 | 1.265-1.310 | 1.506-1.604 | -0.444 | 0.628 | -0.487, -0.401 | 0.502, 0.754 | 0.041 | 0.134 | 0.012, 0.071 | 0.066, 0.201 |
| s | 2012-2014 | 15 | 7,638 | 1.272 | 1.478 | 1.204-1.341 | 1.361-1.595 | -0.399 | 0.518 | -0.546, -0.251 | 0.185, 0.851 | 0.055 | 0.043 | -0.018, 0.128 | -0.093, 0.178 |
| t | 2003-2011 | 77 | 61,788 | 1.226 | 1.405 | 1.197-1.254 | 1.361-1.450 | -0.373 | 0.550 | -0.470, -0.277 | 0.330, 0.769 | -0.052 | 0.038 | -0.088, -0.016 | -0.017, 0.093 |
| u | 1993-1999 | 8 | 9,093 | 1.144 | 1.229 | 1.118-1.170 | 1.170-1.287 | -0.481 | 0.204 | -0.695, -0.267 | -0.604, 1.012 | -0.080 | -0.001 | -0.166, 0.007 | -0.217, 0.215 |

HAZ height-for-age Z-score; SMART, results when SMART flagging procedures are applied; WHO, results when biologically impossible data are excluded.

| **agency** | **dates** | **surveys** | **subjects** | **WAZ Standard deviations** | | | | **WAZ Kurtosis** | | | | **WAZ Skewness** | | | |
| --- | --- | --- | --- | --- | --- | --- | --- | --- | --- | --- | --- | --- | --- | --- | --- |
|  |  |  |  | **SMART** | **WHO** | **SMART** | **WHO** | **SMART** | **WHO** | **SMART** | **WHO** | **SMART** | **WHO** | **SMART** | **WHO** |
|  |  | # | # | mean | mean | **±95% CI** | **±95% CI** | mean | mean | **±95% CI** | **±95% CI** | mean | mean | **±95% CI** | **±95% CI** |
| a | 1999-2014 | 45 | 38,476 | 1.023 | 1.058 | 1.000-1.045 | 1.031-1.084 | -0.015 | 0.415 | -0.079, 0.050 | 0.288, 0.542 | -0.211 | 1.058 | -0.247, -0.175 | 1.031, 1.084 |
| b | 2001-2004 | 6 | 4,475 | 1.143 | 1.216 | 1.060-1.227 | 1.103-1.329 | -0.264 | 0.466 | -0.447, -0.081 | 0.124, 0.808 | -0.127 | 1.216 | -0.215, -0.038 | 1.103, 1.329 |
| c | 2000-2011 | 43 | 33,660 | 1.098 | 1.149 | 1.080-1.115 | 1.129-1.169 | -0.187 | 0.366 | -0.245, -0.129 | 0.211, 0.521 | -0.101 | 1.149 | -0.136, -0.066 | 1.129, 1.169 |
| d | 2005-2014 | 378 | 273,255 | 1.020 | 1.060 | 1.012-1.028 | 1.050-1.069 | -0.029 | 0.461 | -0.051, -0.006 | 0.413, 0.510 | -0.115 | 1.060 | -0.128, -0.102 | 1.050, 1.069 |
| e | 2000-2014 | 35 | 29,036 | 1.015 | 1.059 | 0.979-1.052 | 1.013-1.105 | -0.007 | 0.506 | -0.097, 0.084 | 0.354, 0.657 | -0.166 | 1.059 | -0.220, -0.112 | 1.013, 1.105 |
| f | 2005-2006 | 10 | 9,007 | 1.087 | 1.130 | 1.031-1.142 | 1.062-1.198 | -0.124 | 0.293 | -0.286, 0.038 | 0.094, 0.492 | -0.156 | 1.130 | -0.224, -0.088 | 1.062, 1.198 |
| g | 2000-2008 | 15 | 27,940 | 1.131 | 1.187 | 1.092-1.170 | 1.138-1.235 | -0.222 | 0.245 | -0.308, -0.136 | 0.128, 0.363 | -0.078 | 1.187 | -0.165, 0.010 | 1.138, 1.235 |
| h | 2005-2012 | 15 | 14,039 | 1.004 | 1.053 | 0.964-1.045 | 1.000-1.105 | 0.117 | 0.690 | -0.025, 0.259 | 0.468, 0.911 | -0.225 | 1.053 | -0.304, -0.147 | 1.000, 1.105 |
| i | 1992-2014 | 925 | 706,503 | 1.068 | 1.113 | 1.061-1.075 | 1.104-1.121 | -0.142 | 0.280 | -0.158, -0.125 | 0.245, 0.315 | -0.143 | 1.113 | -0.151, -0.134 | 1.104, 1.121 |
| j | 2000-2010 | 13 | 11,099 | 1.132 | 1.207 | 1.090-1.174 | 1.145-1.268 | -0.221 | 0.302 | -0.314, -0.128 | 0.149, 0.456 | -0.160 | 1.207 | -0.219, -0.100 | 1.145, 1.268 |
| k | 2001-2010 | 28 | 26,096 | 1.140 | 1.204 | 1.110-1.170 | 1.168-1.240 | -0.224 | 0.360 | -0.300, -0.148 | 0.187, 0.534 | -0.170 | 1.204 | -0.207, -0.132 | 1.168, 1.240 |
| L | 1992-2013 | 47 | 34,003 | 1.134 | 1.209 | 1.093-1.175 | 1.153-1.265 | -0.281 | 0.343 | -0.352, -0.211 | 0.173, 0.514 | -0.148 | 1.209 | -0.178, -0.118 | 1.153, 1.265 |
| m | 2012-2014 | 7 | 3,973 | 1.010 | 1.020 | 0.963-1.057 | 0.972-1.069 | 0.010 | 0.140 | -0.210, 0.231 | -0.147, 0.428 | -0.135 | 1.020 | -0.225, -0.044 | 0.972, 1.069 |
| n | 1992-2005 | 50 | 36,005 | 1.141 | 1.221 | 1.117-1.165 | 1.192-1.250 | -0.248 | 0.405 | -0.306, -0.190 | 0.246, 0.564 | -0.128 | 1.221 | -0.160, -0.097 | 1.192, 1.250 |
| o | 2000-2000 | 11 | 8,607 | 1.140 | 1.184 | 1.090-1.190 | 1.129-1.240 | -0.253 | 0.088 | -0.383, -0.122 | -0.112, 0.288 | -0.183 | 1.184 | -0.228, -0.138 | 1.129, 1.240 |
| p | 2001-2007 | 6 | 4,730 | 1.187 | 1.270 | 1.097-1.276 | 1.167-1.373 | -0.325 | 0.187 | -0.452, -0.198 | -0.093, 0.467 | -0.181 | 1.270 | -0.232, -0.130 | 1.167, 1.373 |
| q | 1986-2010 | 47 | 21,072 | 1.156 | 1.234 | 1.129-1.183 | 1.200-1.268 | -0.242 | 0.367 | -0.302, -0.182 | 0.203, 0.531 | -0.138 | 1.234 | -0.174, -0.102 | 1.200, 1.268 |
| r | 1998-2005 | 62 | 52,347 | 1.117 | 1.207 | 1.094-1.139 | 1.173-1.240 | -0.099 | 0.575 | -0.154, -0.043 | 0.482, 0.667 | -0.125 | 1.207 | -0.156, -0.094 | 1.173, 1.240 |
| s | 2012-2014 | 15 | 7,638 | 1.082 | 1.144 | 1.031-1.134 | 1.070-1.217 | -0.128 | 0.404 | -0.272, 0.015 | 0.108, 0.699 | -0.099 | 1.144 | -0.163, -0.036 | 1.070, 1.217 |
| t | 2003-2011 | 77 | 61,788 | 1.092 | 1.143 | 1.071-1.113 | 1.117-1.170 | -0.145 | 0.420 | -0.206, -0.084 | 0.084, 0.757 | -0.057 | 1.143 | -0.094, -0.020 | 1.117, 1.170 |
| u | 1993-1999 | 8 | 9,093 | 0.979 | 1.020 | 0.960-0.998 | 0.981-1.060 | -0.085 | 0.654 | -0.313, 0.142 | -0.088, 1.397 | -0.191 | 1.020 | -0.264, -0.118 | 0.981, 1.060 |

WAZ weight-for-age Z-score; SMART, results when SMART flagging procedures are applied; WHO, results when biologically impossible data are excluded.

| **agency** | **dates** | **surveys** | **subjects** | **MUACZ for age Standard deviations** | | | | **MUACZ for age Kurtosis** | | | | **MUACZ for age Skewness** | | | |
| --- | --- | --- | --- | --- | --- | --- | --- | --- | --- | --- | --- | --- | --- | --- | --- |
|  |  |  |  | **SMART** | **WHO** | **SMART** | **WHO** | **SMART** | **WHO** | **SMART** | **WHO** | **SMART** | **WHO** | **SMART** | **WHO** |
|  |  | # | # | mean | mean | **±95% CI** | **±95% CI** | mean | mean | **±95% CI** | **±95% CI** | mean | mean | **±95% CI** | **±95% CI** |
| a | 1999-2014 | 45 | 38,476 | 0.950 | 0.970 | 0.933-0.966 | 0.951-0.989 | 0.043 | 0.411 | -0.012, 0.099 | 0.274, 0.549 | -0.003 | -0.015 | -0.035, 0.030 | -0.058, 0.027 |
| b | 2001-2004 | 6 | 4,475 | 0.968 | 1.012 | 0.903-1.033 | 0.921-1.103 | 0.061 | 0.642 | -0.084, 0.206 | 0.358, 0.927 | -0.081 | -0.192 | -0.176, 0.013 | -0.385, 0.002 |
| c | 2000-2011 | 43 | 33,660 | 0.973 | 1.001 | 0.950-0.996 | 0.975-1.027 | 0.017 | 0.473 | -0.040, 0.075 | 0.329, 0.616 | -0.055 | -0.139 | -0.098, -0.013 | -0.204, -0.073 |
| d | 2005-2014 | 378 | 273,255 | 0.931 | 0.959 | 0.926-0.937 | 0.952-0.965 | 0.114 | 0.660 | 0.093, 0.135 | 0.607, 0.713 | -0.060 | -0.156 | -0.074, -0.046 | -0.178, -0.135 |
| e | 2000-2014 | 35 | 29,036 | 0.976 | 1.009 | 0.948-1.005 | 0.972-1.045 | -0.012 | 0.438 | -0.071, 0.046 | 0.336, 0.539 | -0.027 | -0.106 | -0.062, 0.009 | -0.168, -0.044 |
| f | 2005-2006 | 10 | 9,007 | 0.968 | 1.004 | 0.926-1.009 | 0.952-1.055 | 0.104 | 0.685 | -0.037, 0.245 | 0.412, 0.959 | -0.027 | -0.128 | -0.080, 0.027 | -0.262, 0.007 |
| g | 2000-2008 | 15 | 27,940 | 1.012 | 1.048 | 0.957-1.066 | 0.985-1.110 | -0.030 | 0.436 | -0.153, 0.094 | 0.226, 0.645 | -0.086 | -0.193 | -0.138, -0.033 | -0.269, -0.117 |
| h | 2005-2012 | 15 | 14,039 | 0.984 | 1.010 | 0.954-1.014 | 0.976-1.044 | 0.019 | 0.412 | -0.008, 0.046 | 0.291, 0.532 | -0.036 | -0.063 | -0.109, 0.037 | -0.174, 0.048 |
| i | 1992-2014 | 925 | 706,503 | 1.000 | 1.036 | 0.994-1.006 | 1.029-1.043 | -0.017 | 0.438 | -0.031, -0.004 | 0.410, 0.467 | -0.101 | -0.182 | -0.109, -0.093 | -0.194, -0.169 |
| j | 2000-2010 | 13 | 11,099 | 0.952 | 0.992 | 0.920-0.984 | 0.950-1.035 | 0.155 | 0.772 | 0.052, 0.257 | 0.472, 1.071 | -0.117 | -0.211 | -0.219, -0.015 | -0.350, -0.073 |
| k | 2001-2010 | 28 | 26,096 | 0.987 | 1.026 | 0.962-1.012 | 0.994-1.058 | 0.088 | 0.638 | 0.028, 0.148 | 0.518, 0.757 | -0.144 | -0.270 | -0.186, -0.102 | -0.333, -0.208 |
| L | 1992-2013 | 47 | 34,003 | 1.013 | 1.059 | 0.978-1.048 | 1.015-1.103 | -0.062 | 0.452 | -0.132, 0.008 | 0.310, 0.594 | -0.128 | -0.238 | -0.165, -0.090 | -0.299, -0.177 |
| m | 2012-2014 | 7 | 3,973 | 1.002 | 1.067 | 0.941-1.063 | 0.978-1.157 | 0.113 | 0.867 | -0.052, 0.278 | 0.349, 1.385 | -0.167 | -0.361 | -0.280, -0.054 | -0.572, -0.151 |
| n | 1992-2005 | 50 | 36,005 | 1.059 | 1.113 | 1.029-1.089 | 1.076-1.149 | -0.143 | 0.449 | -0.214, -0.071 | 0.296, 0.602 | -0.110 | -0.167 | -0.144, -0.076 | -0.242, -0.093 |
| o | 2000-2000 | 11 | 8,607 | 1.044 | 1.088 | 1.013-1.074 | 1.049-1.127 | -0.134 | 0.361 | -0.237, -0.031 | 0.148, 0.574 | -0.099 | -0.203 | -0.143, -0.055 | -0.278, -0.127 |
| p | 2001-2007 | 6 | 4,730 | 1.028 | 1.064 | 0.950-1.105 | 0.968-1.160 | 0.129 | 0.461 | -0.057, 0.316 | 0.315, 0.607 | -0.162 | -0.205 | -0.225, -0.098 | -0.319, -0.091 |
| q | 1986-2010 | 47 | 21,072 | 1.073 | 1.124 | 1.043-1.104 | 1.090-1.157 | -0.113 | 0.434 | -0.187, -0.040 | 0.283, 0.585 | -0.131 | -0.251 | -0.163, -0.099 | -0.308, -0.193 |
| r | 1998-2005 | 62 | 52,347 | 1.032 | 1.084 | 1.008-1.056 | 1.053-1.115 | -0.030 | 0.504 | -0.082, 0.022 | 0.407, 0.600 | -0.116 | -0.242 | -0.147, -0.086 | -0.285, -0.198 |
| s | 2012-2014 | 15 | 7,638 | 1.001 | 1.054 | 0.956-1.045 | 0.987-1.121 | -0.050 | 0.524 | -0.168, 0.069 | 0.127, 0.922 | -0.079 | -0.157 | -0.152, -0.006 | -0.290, -0.025 |
| t | 2003-2011 | 77 | 61,788 | 1.035 | 1.086 | 1.015-1.054 | 1.059-1.114 | -0.058 | 0.461 | -0.122, 0.007 | 0.362, 0.559 | -0.086 | -0.118 | -0.130, -0.042 | -0.173, -0.063 |
| u | 1993-1999 | 8 | 9,093 | 0.910 | 0.931 | 0.881-0.939 | 0.896-0.966 | 0.154 | 0.603 | 0.019, 0.289 | 0.176, 1.030 | -0.125 | -0.238 | -0.176, -0.074 | -0.383, -0.092 |

MUACZ mid-upper arm circumference for age Z-score (all using WHO2006 standards); SMART, results when SMART flagging procedures are applied; WHO, results when biologically impossible data are excluded.

| **agency** | **dates** | **surveys** | **subjects** | **MUACZ for height Standard deviations** | | | | **MUACZ for height Kurtosis** | | | | **MUACZ for height Skewness** | | | |
| --- | --- | --- | --- | --- | --- | --- | --- | --- | --- | --- | --- | --- | --- | --- | --- |
|  |  |  |  | **SMART** | **WHO** | **SMART** | **WHO** | **SMART** | **WHO** | **SMART** | **WHO** | **SMART** | **WHO** | **SMART** | **WHO** |
|  |  | # | # | mean | mean | **±95% CI** | **±95% CI** | mean | mean | **±95% CI** | **±95% CI** | mean | mean | **±95% CI** | **±95% CI** |
| a | 1999-2014 | 45 | 38,476 | 0.939 | 0.957 | 0.922-0.957 | 0.937-0.976 | -0.011 | 0.335 | -0.059, 0.037 | 0.186, 0.483 | 0.018 | -0.013 | -0.010, 0.045 | -0.056, 0.030 |
| b | 2001-2004 | 6 | 4,475 | 0.939 | 0.973 | 0.877-1.001 | 0.887-1.059 | 0.054 | 0.582 | -0.103, 0.211 | 0.212, 0.952 | -0.087 | -0.198 | -0.137, -0.037 | -0.367, -0.030 |
| c | 2000-2011 | 43 | 33,660 | 0.940 | 0.965 | 0.918-0.962 | 0.941-0.989 | 0.005 | 0.494 | -0.056, 0.066 | 0.326, 0.663 | -0.057 | -0.152 | -0.101, -0.013 | -0.219, -0.085 |
| d | 2005-2014 | 378 | 273,255 | 0.903 | 0.925 | 0.898-0.909 | 0.919-0.931 | 0.117 | 0.595 | 0.096, 0.139 | 0.539, 0.651 | -0.057 | -0.141 | -0.071, -0.044 | -0.161, -0.121 |
| e | 2000-2014 | 35 | 29,036 | 0.962 | 0.992 | 0.937-0.988 | 0.959-1.025 | -0.012 | 0.437 | -0.059, 0.036 | 0.306, 0.569 | -0.014 | -0.112 | -0.050, 0.022 | -0.169, -0.054 |
| f | 2005-2006 | 10 | 9,007 | 0.956 | 0.993 | 0.923-0.989 | 0.949-1.037 | 0.040 | 0.750 | -0.133, 0.213 | 0.314, 1.186 | -0.03 | -0.135 | -0.089, 0.030 | -0.303, 0.032 |
| g | 2000-2008 | 15 | 27,940 | 0.973 | 1.002 | 0.924-1.022 | 0.947-1.057 | -0.014 | 0.467 | -0.135, 0.107 | 0.213, 0.721 | -0.079 | -0.182 | -0.121, -0.038 | -0.260, -0.105 |
| h | 2005-2012 | 15 | 14,039 | 0.979 | 1.000 | 0.949-1.009 | 0.967-1.032 | -0.033 | 0.299 | -0.078, 0.011 | 0.200, 0.399 | -0.025 | -0.037 | -0.091, 0.041 | -0.129, 0.055 |
| i | 1992-2014 | 925 | 706,503 | 0.975 | 1.005 | 0.969-0.981 | 0.998-1.012 | 0.001 | 0.438 | -0.013, 0.014 | 0.408, 0.468 | -0.100 | -0.182 | -0.109, -0.092 | -0.194, -0.169 |
| j | 2000-2010 | 13 | 11,099 | 0.936 | 0.967 | 0.909-0.963 | 0.926-1.009 | 0.174 | 0.633 | 0.035, 0.312 | 0.328, 0.938 | -0.122 | -0.178 | -0.218, -0.026 | -0.315, -0.040 |
| k | 2001-2010 | 28 | 26,096 | 0.956 | 0.989 | 0.934-0.979 | 0.960-1.018 | 0.079 | 0.636 | 0.009, 0.148 | 0.477, 0.796 | -0.117 | -0.243 | -0.160, -0.073 | -0.311, -0.174 |
| L | 1992-2013 | 47 | 34,003 | 0.988 | 1.030 | 0.956-1.020 | 0.989-1.071 | -0.060 | 0.496 | -0.122, 0.002 | 0.332, 0.660 | -0.128 | -0.243 | -0.165, -0.090 | -0.309, -0.176 |
| m | 2012-2014 | 7 | 3,973 | 0.992 | 1.049 | 0.927-1.056 | 0.957-1.141 | 0.190 | 0.917 | 0.044, 0.336 | 0.382, 1.453 | -0.177 | -0.365 | -0.283, -0.070 | -0.593, -0.137 |
| n | 1992-2005 | 50 | 36,005 | 1.033 | 1.080 | 1.003-1.064 | 1.043-1.118 | -0.103 | 0.508 | -0.171, -0.035 | 0.311, 0.704 | -0.103 | -0.160 | -0.139, -0.067 | -0.238, -0.082 |
| o | 2000-2000 | 11 | 8,607 | 1.038 | 1.079 | 1.007-1.070 | 1.041-1.116 | -0.108 | 0.402 | -0.218, 0.003 | 0.164, 0.640 | -0.118 | -0.200 | -0.188, -0.048 | -0.301, -0.100 |
| p | 2001-2007 | 6 | 4,730 | 0.990 | 1.023 | 0.924-1.057 | 0.936-1.109 | 0.149 | 0.429 | 0.025, 0.273 | 0.322, 0.535 | -0.141 | -0.178 | -0.207, -0.076 | -0.282, -0.074 |
| q | 1986-2010 | 47 | 21,072 | 1.042 | 1.087 | 1.014-1.070 | 1.054-1.120 | -0.065 | 0.479 | -0.141, 0.011 | 0.318, 0.640 | -0.137 | -0.247 | -0.171, -0.102 | -0.307, -0.188 |
| r | 1998-2005 | 62 | 52,347 | 1.011 | 1.055 | 0.985-1.038 | 1.022-1.089 | 0.013 | 0.525 | -0.042, 0.069 | 0.419, 0.631 | -0.124 | -0.235 | -0.153, -0.096 | -0.281, -0.189 |
| s | 2012-2014 | 15 | 7,638 | 0.985 | 1.037 | 0.942-1.027 | 0.965-1.108 | -0.041 | 0.501 | -0.162, 0.080 | 0.099, 0.904 | -0.076 | -0.122 | -0.147, -0.004 | -0.258, 0.013 |
| t | 2003-2011 | 77 | 61,788 | 1.021 | 1.070 | 1.000-1.042 | 1.043-1.098 | -0.025 | 0.523 | -0.091, 0.041 | 0.414, 0.631 | -0.101 | -0.145 | -0.145, -0.056 | -0.200, -0.089 |
| u | 1993-1999 | 8 | 9,093 | 0.903 | 0.923 | 0.878-0.929 | 0.895-0.952 | 0.095 | 0.528 | -0.023, 0.213 | 0.151, 0.905 | -0.089 | -0.185 | -0.170, -0.009 | -0.322, -0.048 |

MUACZ mid-upper arm circumference for height Z-score (all using WHO2006 standards); SMART, results when SMART flagging procedures are applied; WHO, results when biologically impossible data are excluded.
