# Supplementary material for: Change in quality of malnutrition surveys between 1986 and 2015
Source: Emerg Themes Epidemiol. 2018 May 28;15:8. doi: 10.1186/s12982-018-0075-9 (PMC5972441; doi:10.1186/s12982-018-0075-9)
Supplement: Supplementary file 2 — Additional file 2. Plots of individual survey’s anthropometric variables. All survey data except for that contributed by agency “t”. XY plots of the individual survey SDs against the actual date of the survey for respectively: WHZ, HAZ, MUAC-for-age, WAZ, MUAC-for-height, absolute MUAC (each applying SMART and WHO flags), chronological age, absolute weight and absolute height. The polynomial regression lines are shown in red. [file 12982_2018_75_MOESM2_ESM.pdf]

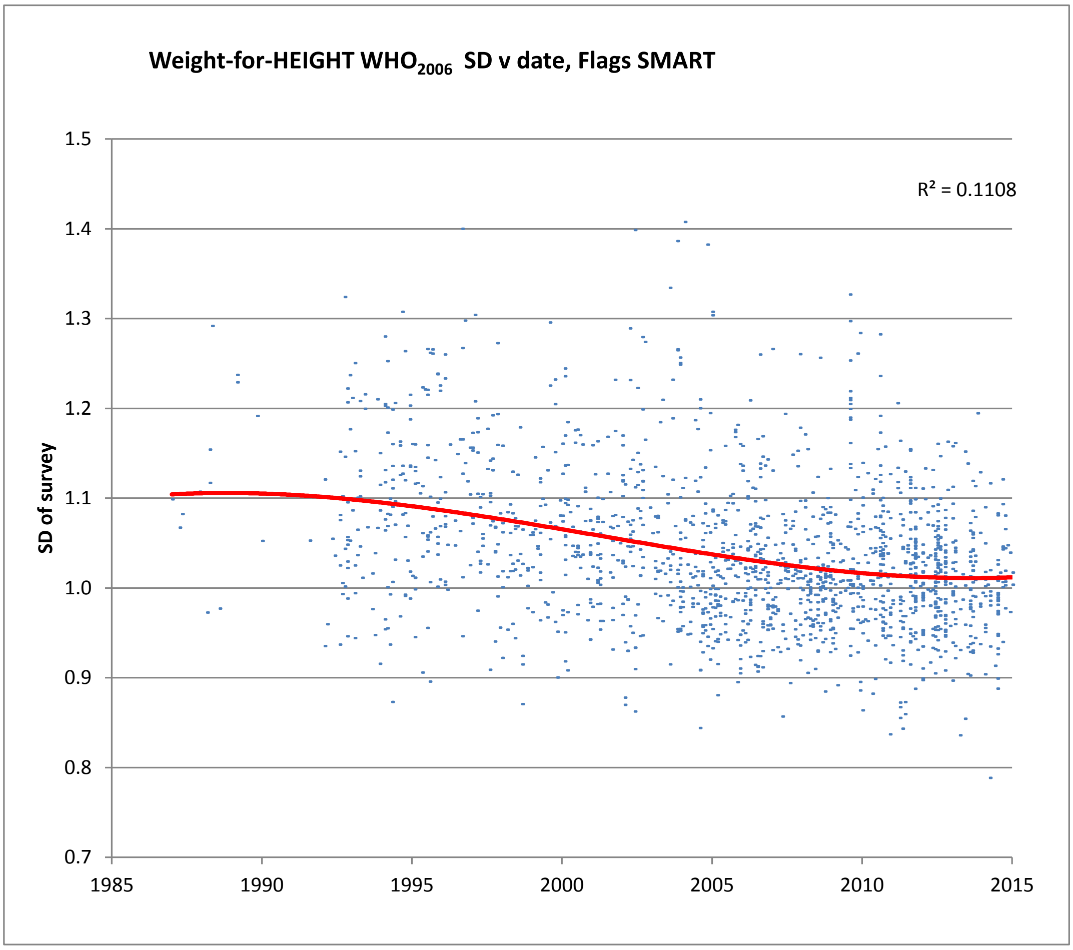

Weight-for-HEIGHT WHO2006 SD v date, Flags SMART
R² = 0.1108
SD of survey
1.5
1.4
1.3
1.2
1.1
1.0
0.9
0.8
0.7
1985
1990
1995
2000
2005
2010
2015

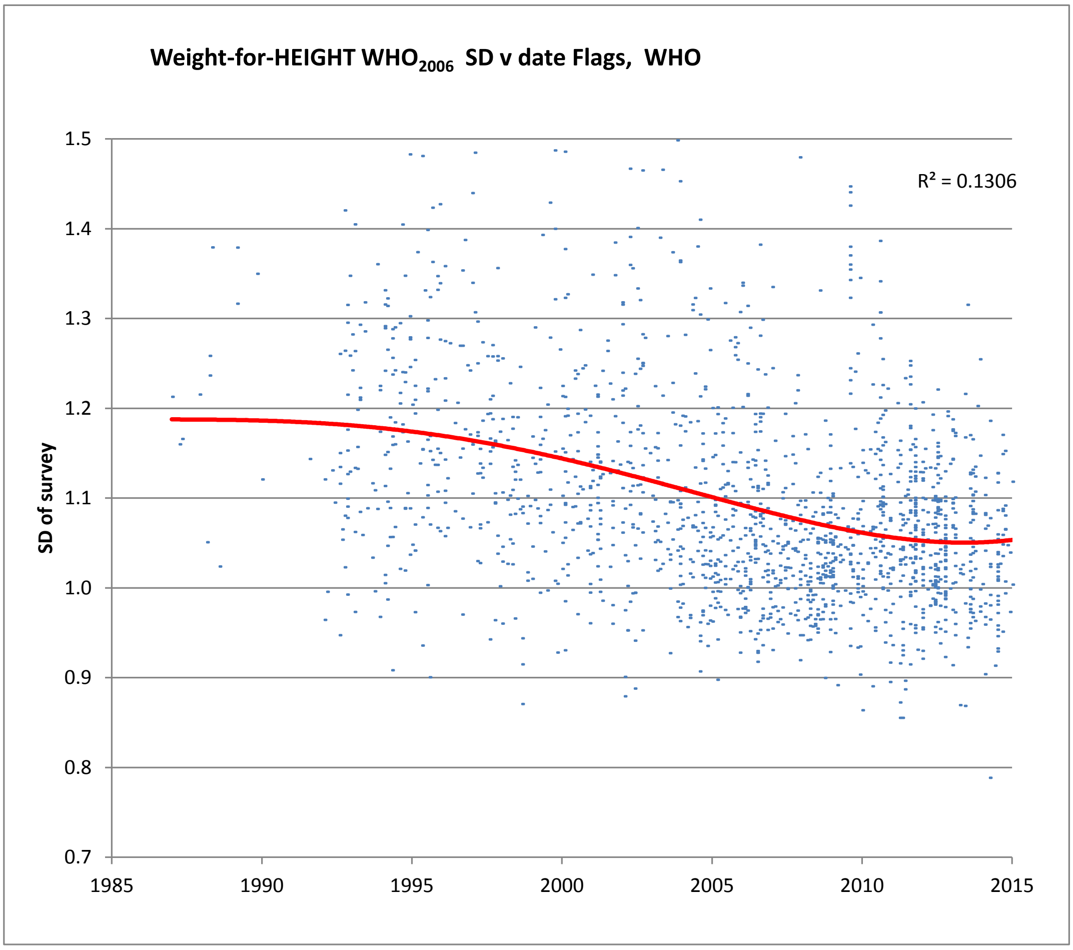

Weight-for-HEIGHT WHO2006 SD v date Flags, WHO
R² = 0.1306
SD of survey
1.5
1.4
1.3
1.2
1.1
1.0
0.9
0.8
0.7
1985
1990
1995
2000
2005
2010
2015

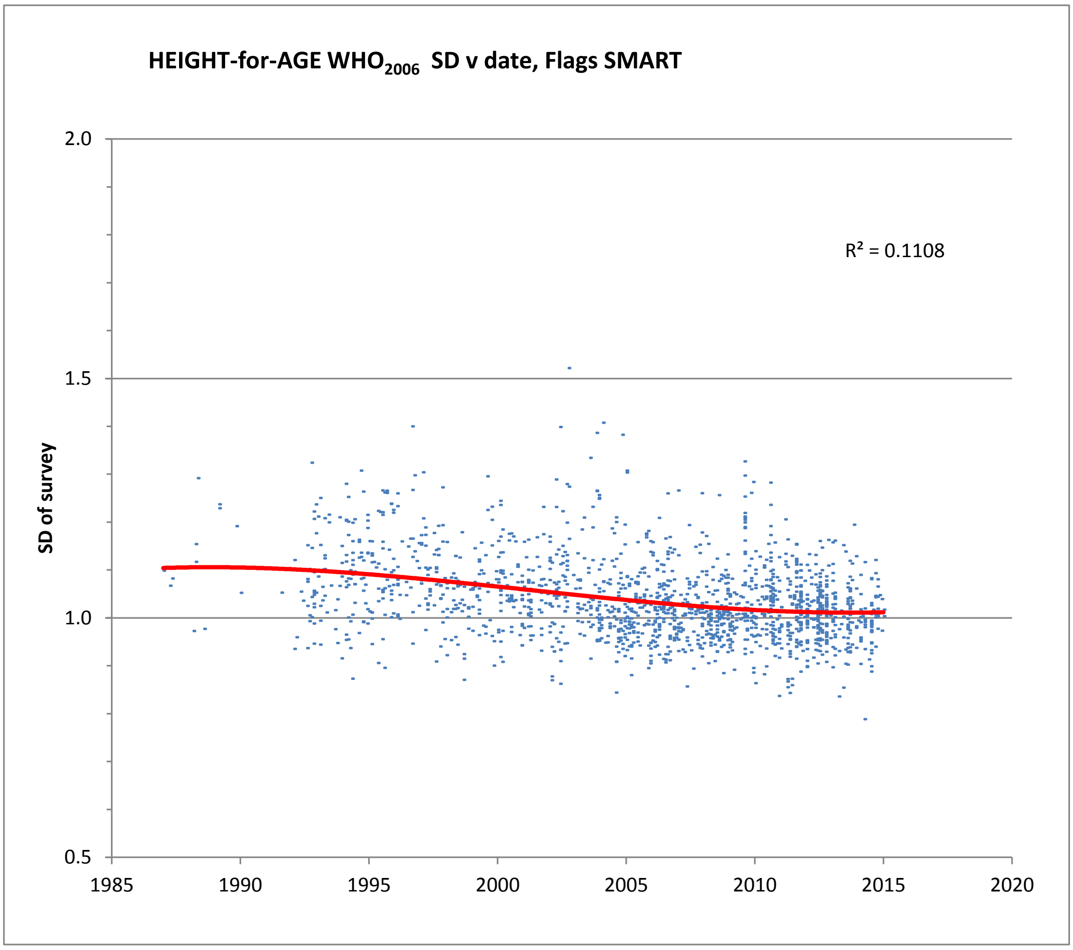
HEIGHT-for-AGE WHO2006 SD v date, Flags SMART
2.0
1.5
1.0
0.5
SD of survey
R² = 0.1108
1985
1990
1995
2000
2005
2010
2015
2020

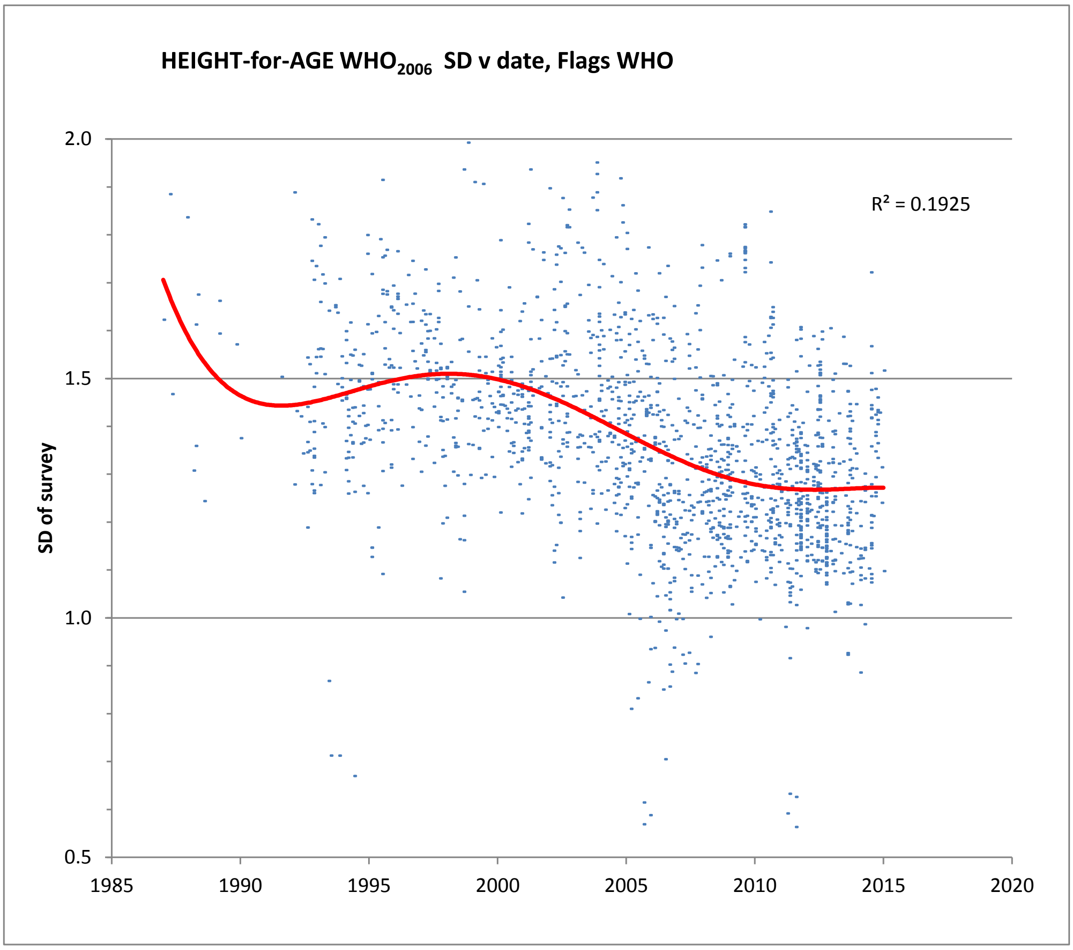
HEIGHT-for-AGE WHO2006 SD v date, Flags WHO
2.0
1.5
1.0
0.5
SD of survey
R² = 0.1925
1985
1990
1995
2000
2005
2010
2015
2020

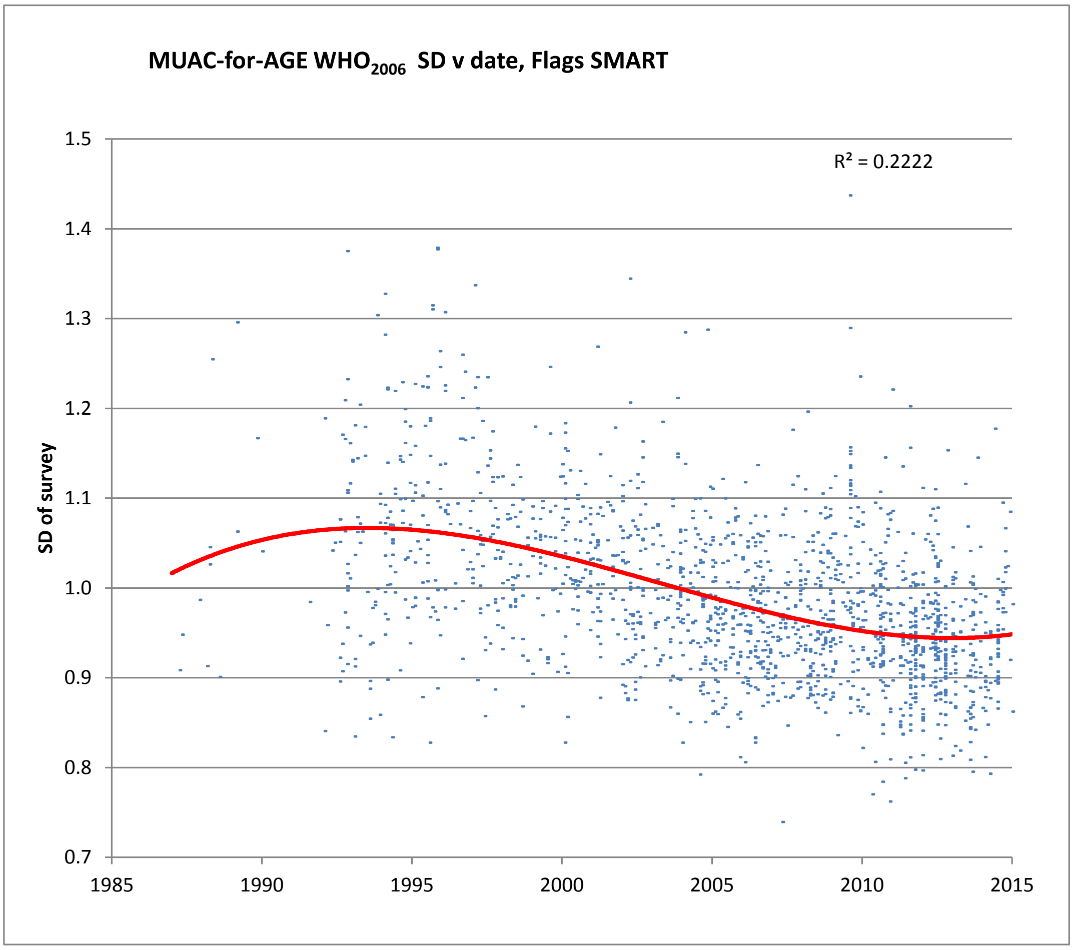

MUAC-for-AGE WHO_{2006} SD v date, Flags SMART
R² = 0.2222
SD of survey
1.5
1.4
1.3
1.2
1.1
1.0
0.9
0.8
0.7
1985
1990
1995
2000
2005
2010
2015

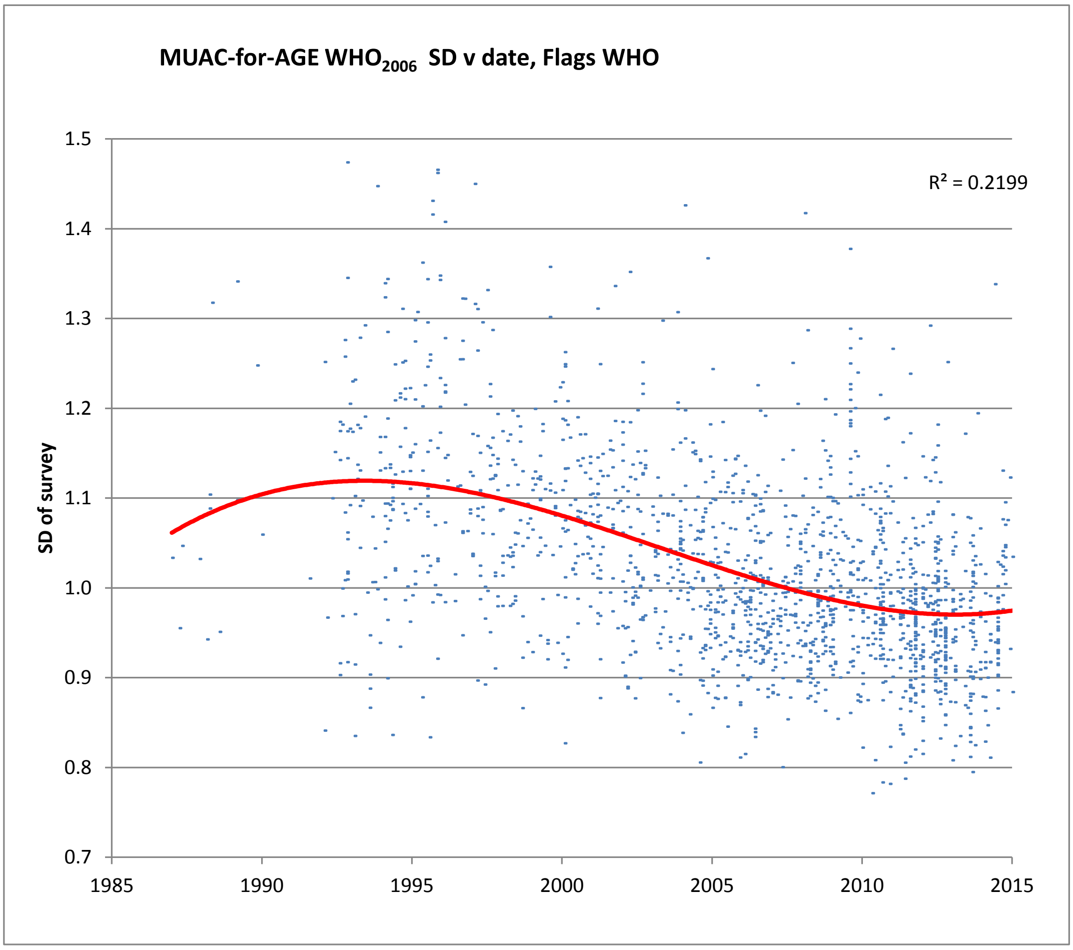

MUAC-for-AGE WHO_{2006} SD v date, Flags WHO
R² = 0.2199
SD of survey
1.5
1.4
1.3
1.2
1.1
1.0
0.9
0.8
0.7
1985
1990
1995
2000
2005
2010
2015

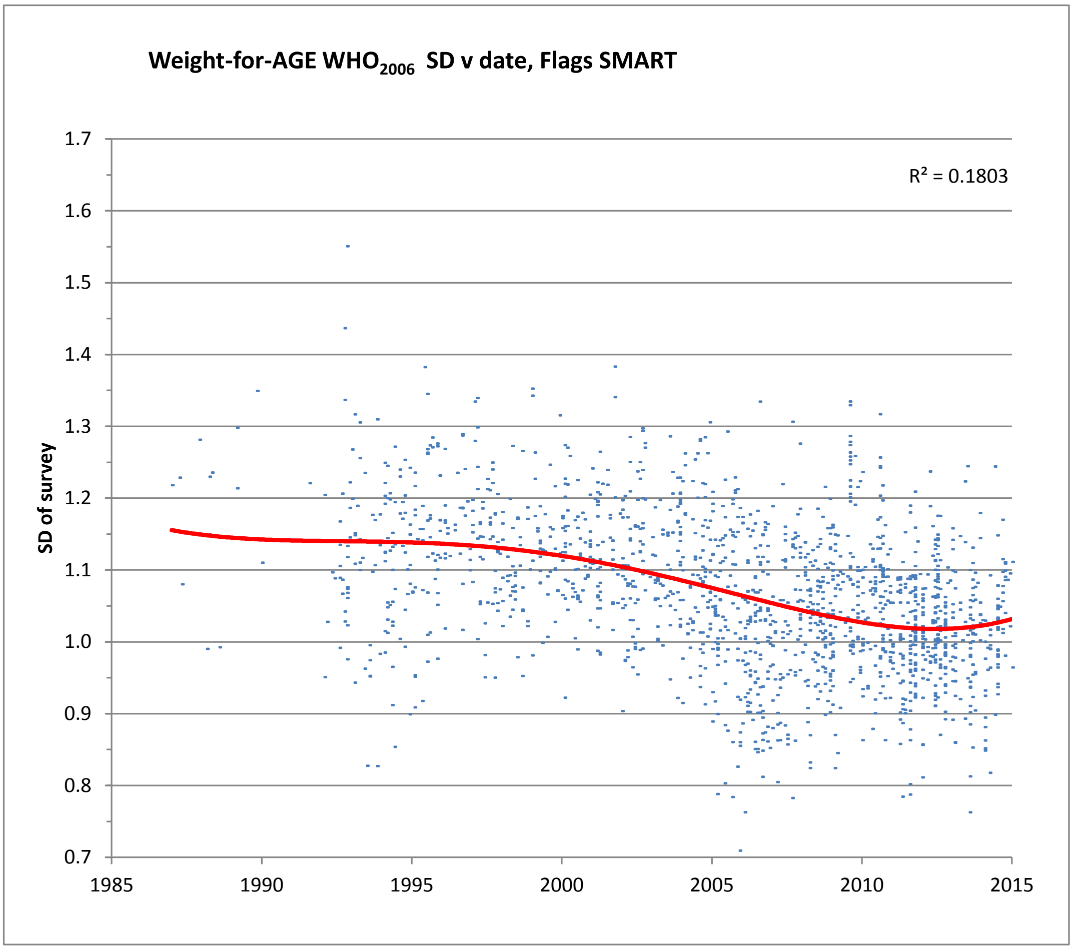
Weight-for-AGE WHO2006 SD v date, Flags SMART
R² = 0.1803
SD of survey
1.7
1.6
1.5
1.4
1.3
1.2
1.1
1.0
0.9
0.8
0.7
1985
1990
1995
2000
2005
2010
2015

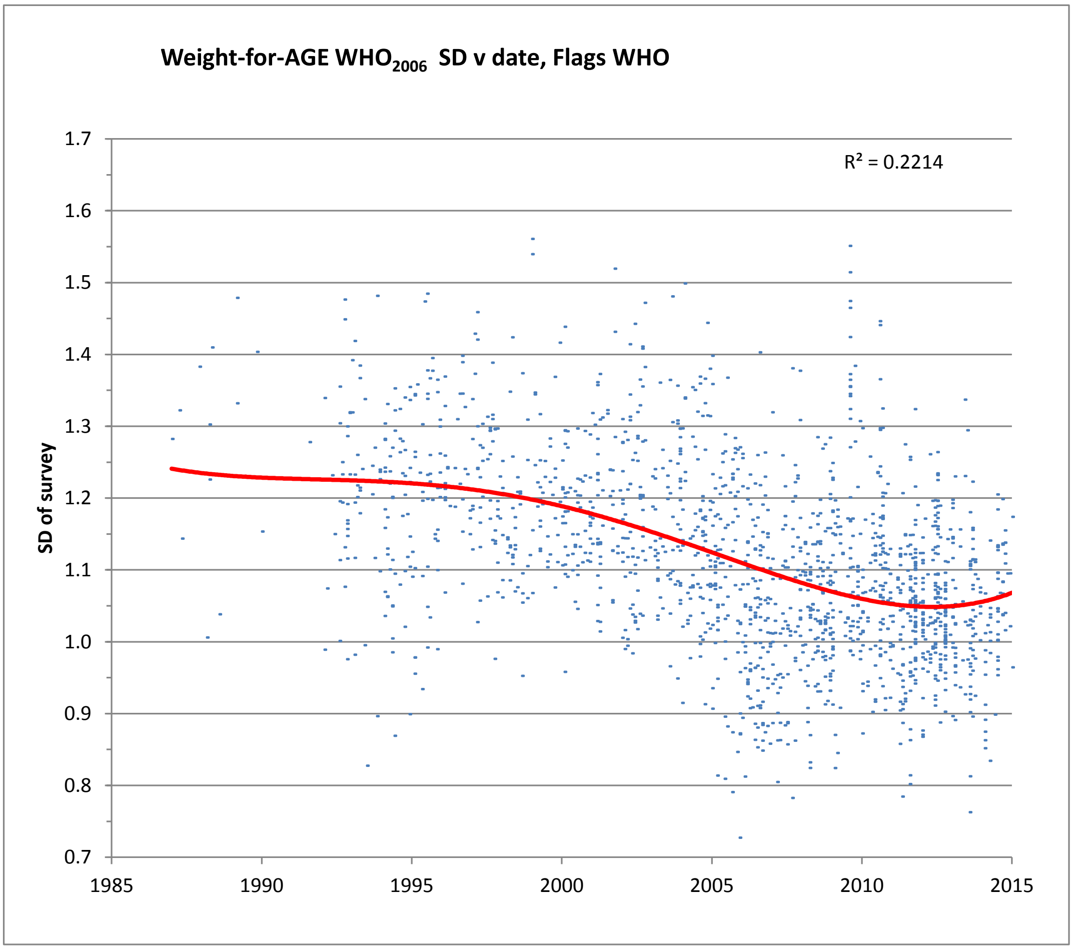
Weight-for-AGE WHO2006 SD v date, Flags WHO
R² = 0.2214
SD of survey
1.7
1.6
1.5
1.4
1.3
1.2
1.1
1.0
0.9
0.8
0.7
1985
1990
1995
2000
2005
2010
2015

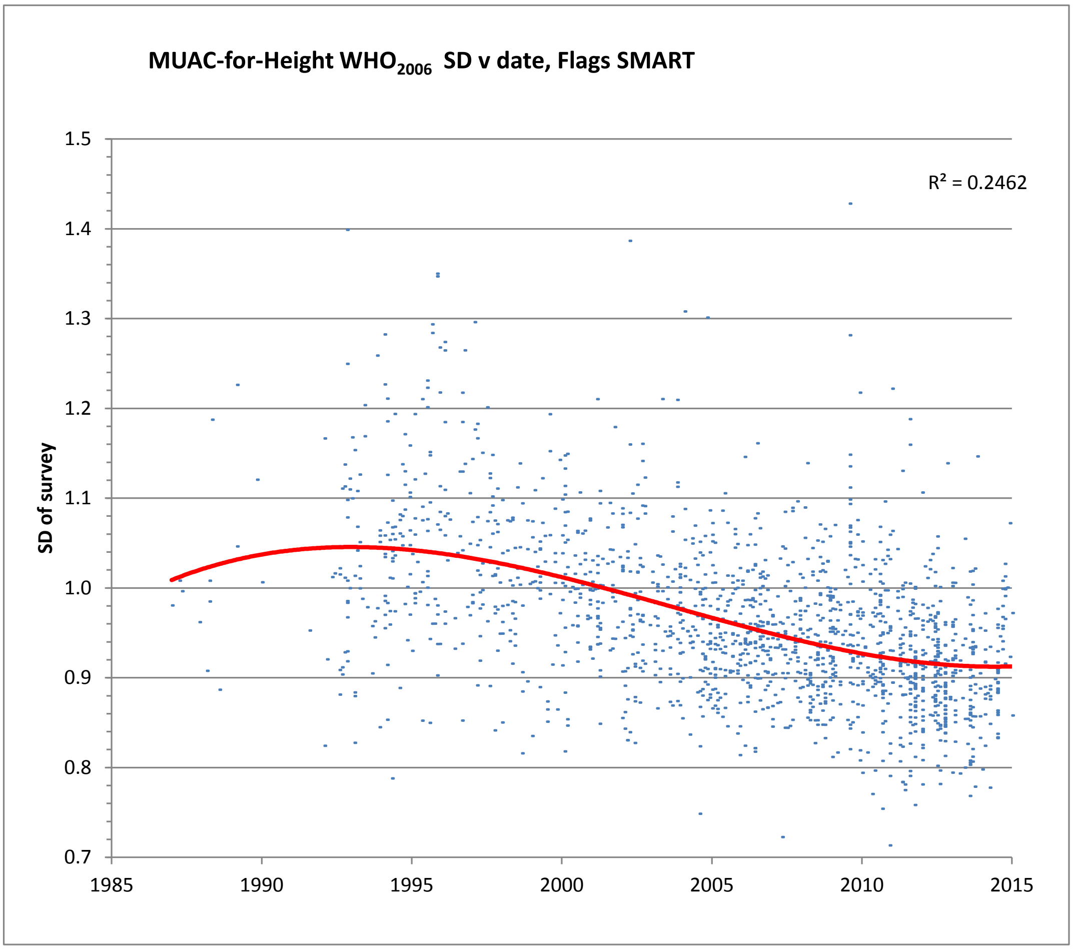
MUAC-for-Height WHO_{2006} SD v date, Flags SMART
$R^2 = 0.2462$
SD of survey
1.5
1.4
1.3
1.2
1.1
1.0
0.9
0.8
0.7
1985
1990
1995
2000
2005
2010
2015

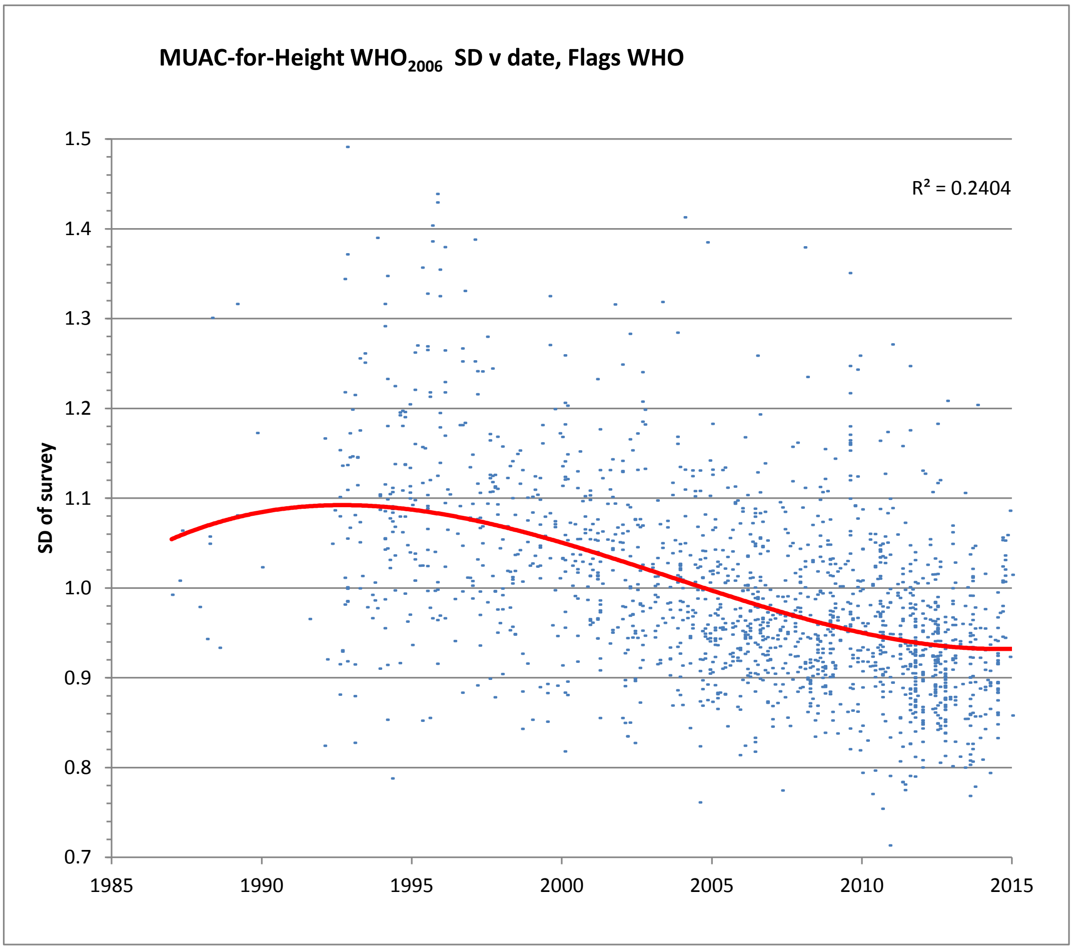
MUAC-for-Height WHO_{2006} SD v date, Flags WHO
$R^2 = 0.2404$
SD of survey
1.5
1.4
1.3
1.2
1.1
1.0
0.9
0.8
0.7
1985
1990
1995
2000
2005
2010
2015

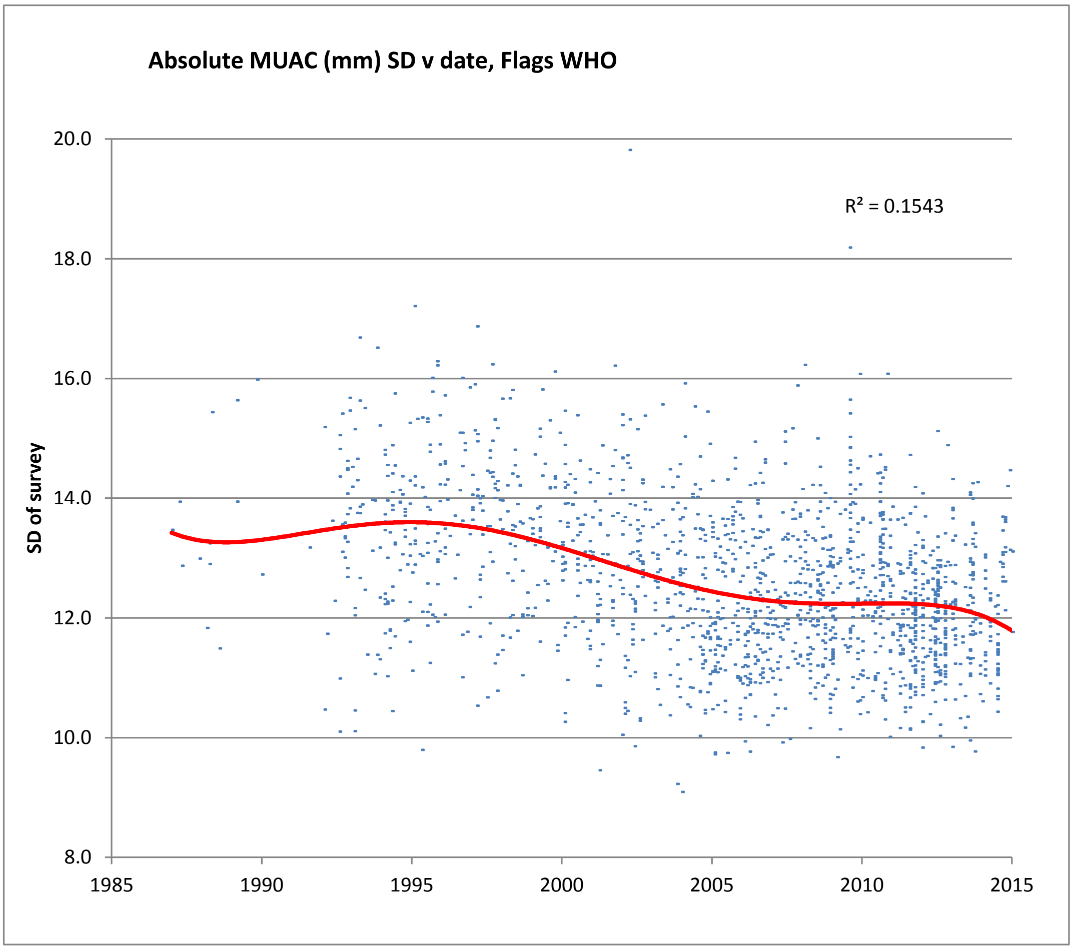
Absolute MUAC (mm) SD v date, Flags WHO
R² = 0.1543
SD of survey
20.0
18.0
16.0
14.0
12.0
10.0
8.0
1985
1990
1995
2000
2005
2010
2015

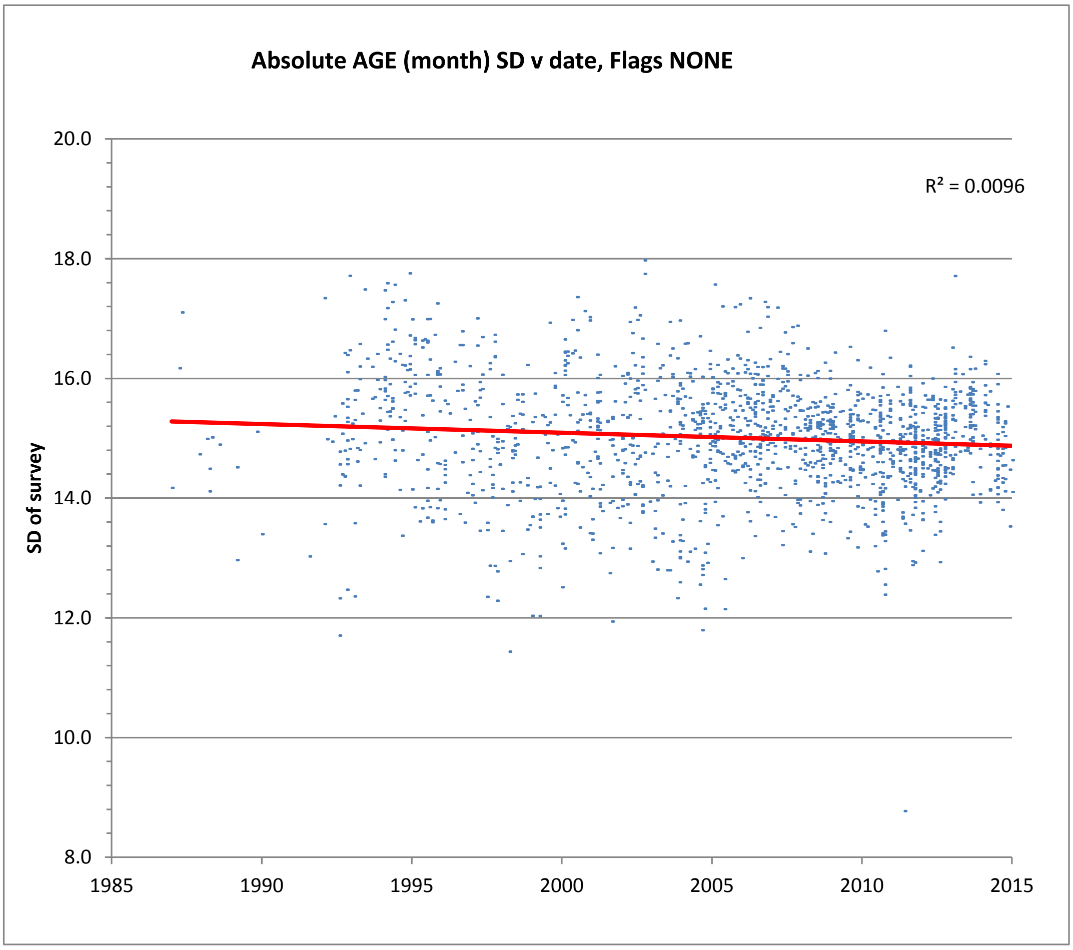
Absolute AGE (month) SD v date, Flags NONE
R² = 0.0096
SD of survey
20.0
18.0
16.0
14.0
12.0
10.0
8.0
1985
1990
1995
2000
2005
2010
2015

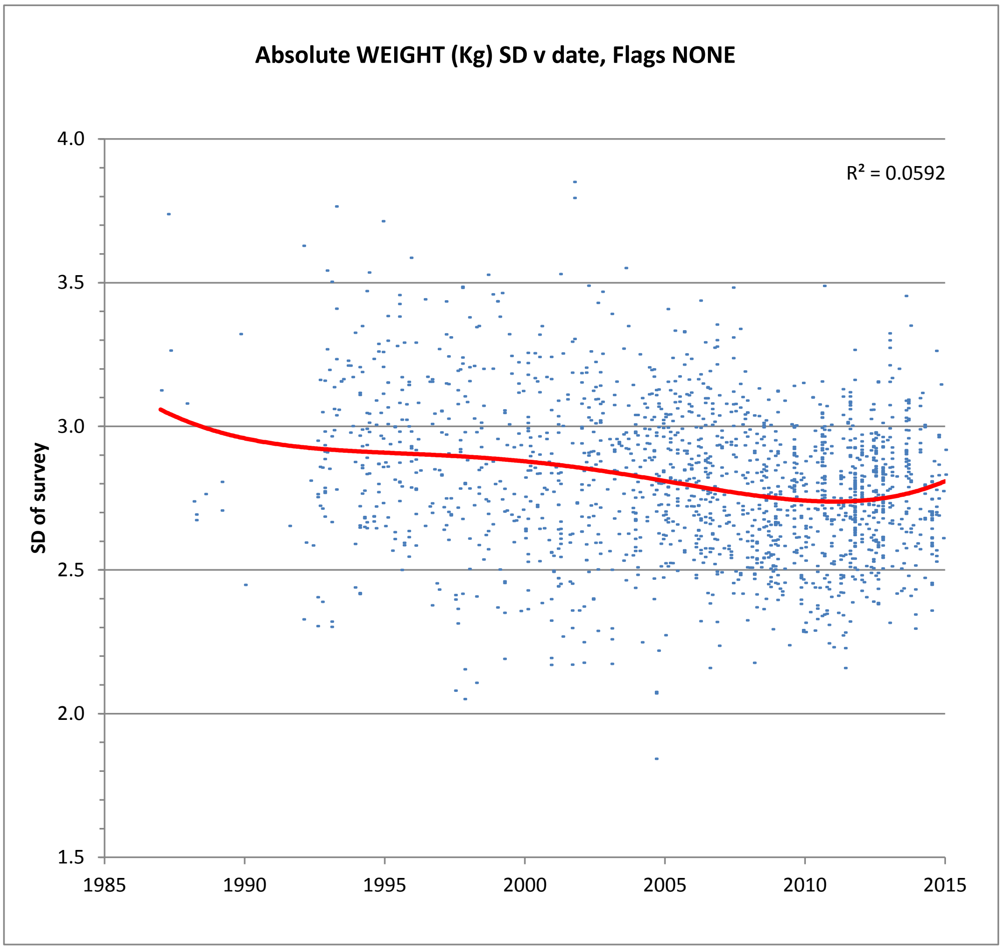
Absolute WEIGHT (Kg) SD v date, Flags NONE
R² = 0.0592
SD of survey
4.0
3.5
3.0
2.5
2.0
1.5
1985
1990
1995
2000
2005
2010
2015

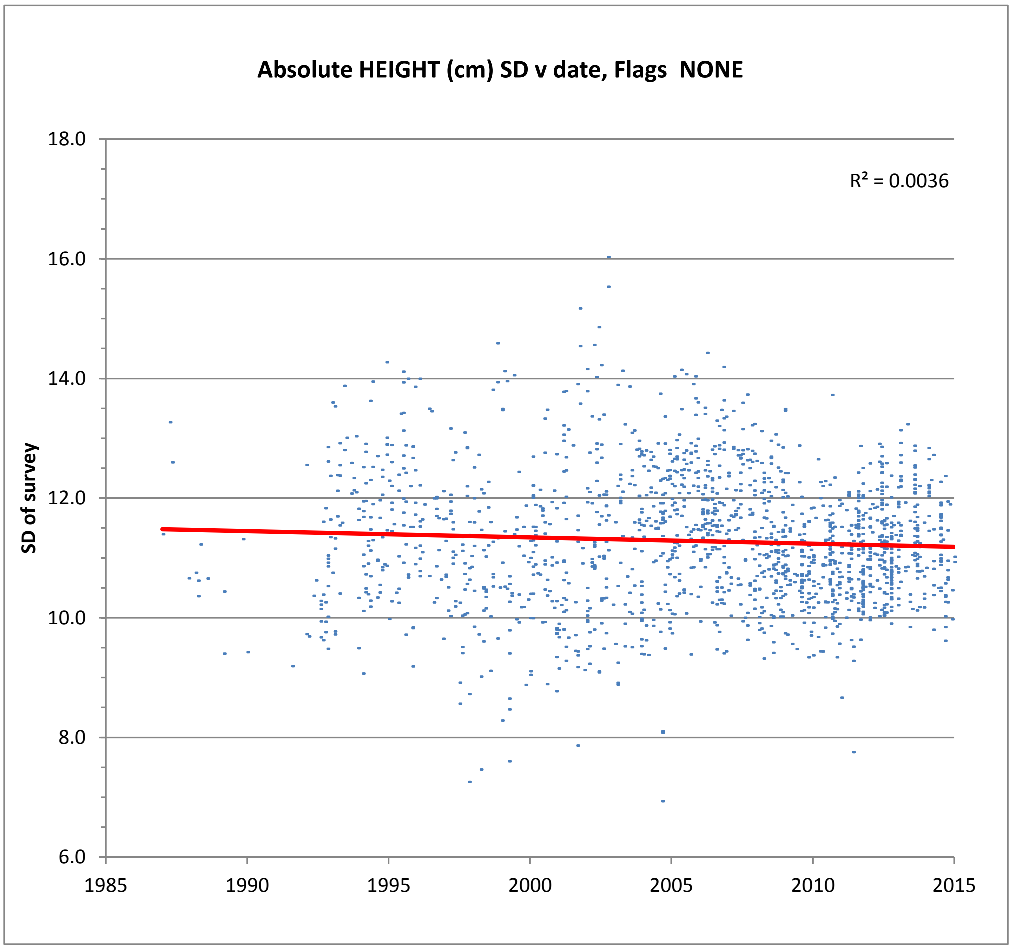
Absolute HEIGHT (cm) SD v date, Flags NONE
R² = 0.0036
SD of survey
18.0
16.0
14.0
12.0
10.0
8.0
6.0
1985
1990
1995
2000
2005
2010
2015
